# Deletion of the N-Terminal Domain of Yeast Eukaryotic Initiation Factor 4B Reprograms Translation and Reduces Growth in Urea

**DOI:** 10.3389/fmolb.2021.787781

**Published:** 2022-01-03

**Authors:** Xiaozhuo Liu, Houtan Moshiri, Qian He, Ansuman Sahoo, Sarah E. Walker

**Affiliations:** Department of Biological Sciences, SUNY at Buffalo, Buffalo, NY, United States

**Keywords:** eukaryotic translation, translation initiation, eIF4B, ribosome, mRNA control

## Abstract

The yeast eukaryotic initiation factor 4B binds the 40S subunit in translation preinitiation complexes (PICs), promoting mRNA recruitment. Recent evidence indicates yeast mRNAs have variable dependence on eIF4B under optimal growth conditions. Given the ability of eIF4B to promote translation as a function of nutrient conditions in mammalian cells, we wondered if eIF4B activities in translation could alter phenotypes in yeast through differential mRNA selection for translation. Here we compared the effects of disrupting yeast eIF4B RNA- and 40S-binding motifs under ∼1400 growth conditions. The RNA-Recognition Motif (RRM) was dispensable for stress responses, but the 40S-binding N-terminal Domain (NTD) promoted growth in response to stressors requiring robust cellular integrity. In particular, the NTD conferred a strong growth advantage in the presence of urea, which may be important for pathogenesis of related fungal species. Ribosome profiling indicated that similar to complete eIF4B deletion, deletion of the NTD dramatically reduced translation, particularly of those mRNAs with long and highly structured 5-prime untranslated regions. This behavior was observed both with and without urea exposure, but the specific mRNA pool associated with ribosomes in response to urea differed. Deletion of the NTD led to relative increases in ribosome association of shorter transcripts with higher dependence on eIF4G, as was noted previously for eIF4B deletion. Gene ontology analysis indicated that proteins encoded by eIF4B NTD-dependent transcripts were associated with the cellular membrane system and the cell wall, while NTD-independent transcripts encoded proteins associated with cytoplasmic proteins and protein synthesis. This analysis highlighted the difference in structure content of mRNAs encoding membrane versus cytoplasmic housekeeping proteins and the variable reliance of specific gene ontology classes on various initiation factors promoting otherwise similar functions. Together our analyses suggest that deletion of the eIF4B NTD prevents cellular stress responses by affecting the capacity to translate a diverse mRNA pool.

## Introduction

Translation initiation begins with the formation of a translation preinitiation complex (PIC) comprised of an initiator Met-tRNA•eIF2•GTP ternary complex bound to the 40S ribosomal subunit along with eIFs 1, 1A, 5 and the multisubunit eIF3. Simultaneously, mRNAs are complexed with the eIF4F complex and Ded1/Ddx3, which are proposed to unwind secondary structure and promote PIC binding to mRNA. The eIF4F complex is comprised of three factors. eIF4E binds the 5′cap structure and eIF4G. eIF4G acts as a scaffold linking the cap to the helicase, and as such binds to mRNAs, eIF4E, eIF4A, and other proteins. eIF4A is a DEAD-box RNA helicase with activity modulated by changes in conformation upon binding to RNA, eIF4G, eIF4B, and in mammalian cells, eIF4H (Mitchell et al., 2011). This complex is thought to serve multiple purposes: *1*) interactions of the 5′cap bound to eIF4E with other components of the PIC bound to eIF4G direct PIC loading to the 5′end of mRNAs, and *2*) helicase activity of eIF4A melts mRNA secondary structure near the cap and throughout the 5-prime untranslated region (5′UTR) to allow effective loading at the cap and scanning through 5′UTRs. The associated protein eIF4B promotes the activity of the eIF4F complex (Rozovsky et al., 2008; Özeş et al., 2011).

A number of observations indicate the importance of eIF4B in translating structured mRNAs and promoting the activity of eIF4A/eIF4F both *in vitro* and *in vivo* (Dmitriev et al., 2003; Mitchell et al., 2010; Sen et al., 2016). In fact, eIF4B in yeast was first discovered by two groups as both a multicopy suppressor of a temperature-sensitive eIF4A mutation, as well as a protein that interacted with antibodies against the 5′-cap complex (Altmann et al., 1993; Coppolecchia et al., 1993). This indicates important functional interaction between eIF4F and eIF4B. One model for eIF4B function suggests that eIF4B enhances eIF4A mRNA helicase activity at the step of mRNA activation or scanning to allow structured mRNA translation (Rogers et al., 2001; Özeş et al., 2011; Andreou and Klostermeier, 2014). However, recent work indicates that some classes of mRNAs have a hyperdependence on eIF4B while showing less relative dependence on eIF4A. This could suggest eIF4B performs both eIF4A-dependent and eIF4A/eIF4F-independent activities during translation, or that functions of eIF4A are universally important for translation (Park et al., 2011; Sen et al., 2015; Sen et al., 2016). These eIF4A-independent functions could stem from the ability of eIF4B to bind to the 40S subunit and promote conformational changes in the mRNA binding channel (Walker et al., 2013; Sen et al., 2016).

**FIGURE 1:**
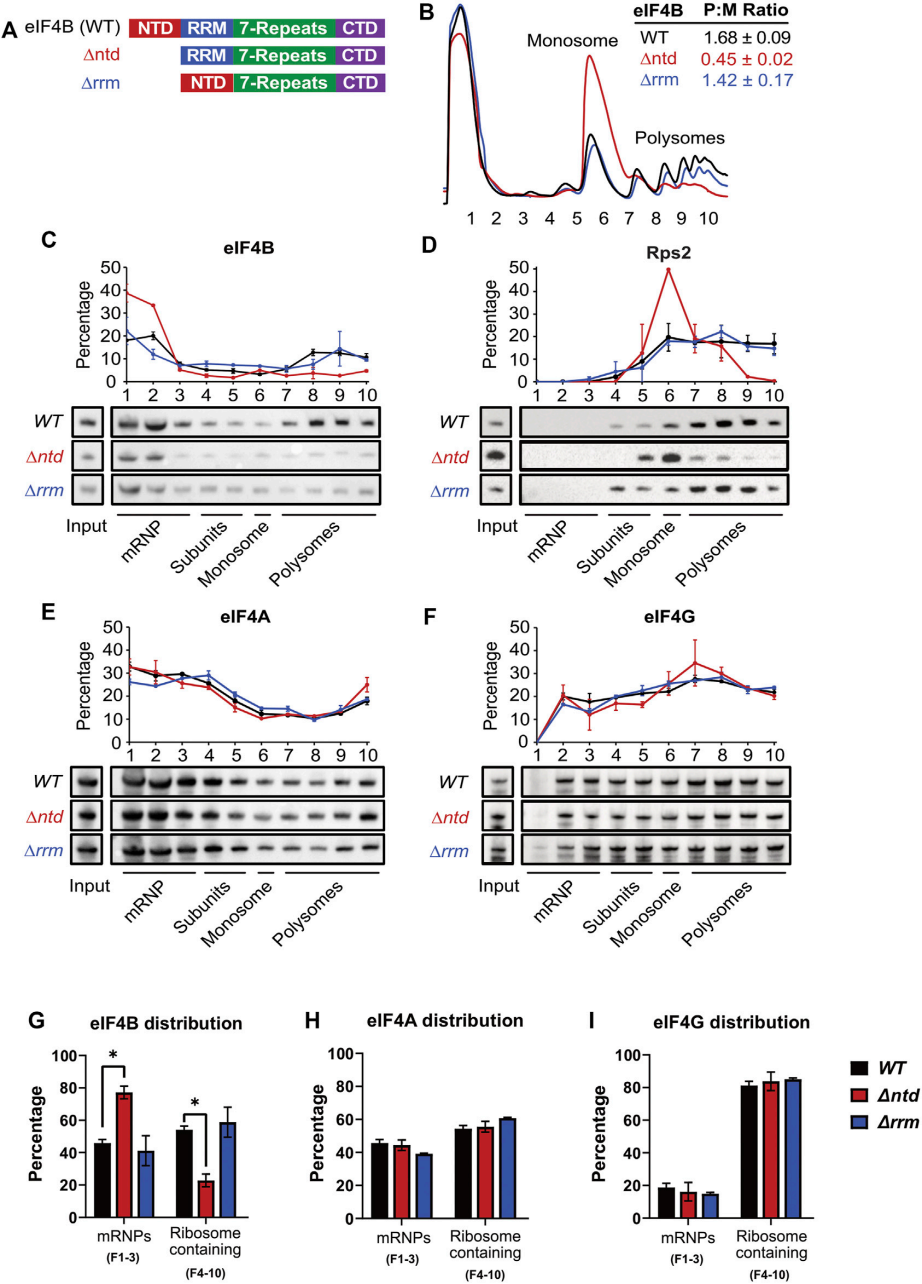
The N-terminal domain of eIF4B binds the ribosome and promotes translation in a prototrophic yeast strain. **(A)** Schematic of eIF4B functional domains in WT, ∆ntd, and ∆rrm constructs. **(B)** Lysates from strains harboring WT (black), ∆ntd (red), or ∆rrm (blue) eIF4B were fractionated on 5–45% sucrose gradients and monitored by absorbance at 254 nm. Polysome to monosome (P:M) ratios were calculated by quantifying the area under the curve for the indicated monosome and polysome peaks. The Mean P:M ratios from three or more biological replicates (representative traces shown) are reported ± standard error of the mean. **(C**–**F)** Lysates were crosslinked with 1% formaldehyde prior to running on 5–45% sucrose gradients. The distributions of eIF4B **(C)**, Rps2 (**D**, an indicator of 40S subunits/monosomes/polysomes), eIF4A **(E)**, and eIF4G **(F)** in mRNP, subunit, monosome, and polysome fractions were determined by western blotting indicated gradient fractions. The line graphs show the mean percentages of total indicated protein (quantified across the gradient) that was present in each fraction. **(G**–**I)** The distributions of eIF4B **(G)**, eIF4A **(H)** and eIF4G **(I)** in mRNP vs. ribosome containing fractions were analyzed by an unpaired students *t* test. The only significant differences observed were for ∆ntd versus WT eIF4B distributions. Asterisk indicates *p* value ≤0.05.

Yeast eIF4B can be divided into four functional domains: an N-terminal domain (NTD), an RNA-recognition motif (RRM), a 7-repeats domain, and a C-terminal domain (Figure 1A; (Altmann et al., 1993; Coppolecchia et al., 1993). While the RRM has a defined globular structure with conserved RNA-binding motifs (Fleming et al., 2003), the other three domains are predicted to be disordered (by analysis of the S288C eIF4B sequence in PONDR (Xue et al., 2010)). Our previous work demonstrated that eIF4B binds directly to the 40S subunit using both the NTD and 7-repeats domains of the protein, independently of the RRM (Walker et al., 2013). The 7-repeats domain consists of imperfect repetitions of a ∼26 amino acid motif that can also bind directly to single-stranded RNA (Altmann et al., 1993; Coppolecchia et al., 1993; Niederberger et al., 1998; Walker et al., 2013). Binding the 40S by either the NTD or 7-repeats domain promotes the movement of a ribosomal protein, Rps20/uS10 (Walker et al., 2013). This induces changes in the conformation of multiple rRNA residues on both the solvent and subunit interfaces of the 40S near domains of Rps20/uS10 that reach into the mRNA binding channel. Both the NTD and the 7-repeats domain of eIF4B enhance its affinity to the 40S and are required for robust mRNA recruitment to the PIC, suggesting these ribosome binding activities are critical for translation. Deletion of either domain resulted in decreased rates of mRNA recruitment and translation. However, the defect conferred by deleting the seven repeats was partially rescued by increasing the concentration of the ∆7-repeats variant *in vitro* or overexpressing the ∆7-repeats protein in cells. The heightened concentration of the ∆7-repeats mutant required for maximal rate suggests the repeats must interact with the 40S or another binding partner for maximal affinity and activity. In contrast, increasing concentrations of the ∆ntd protein did not rescue the decreased rate, suggesting the NTD affects the mechanism by which eIF4B accelerates mRNA recruitment (Walker et al., 2013). Deletion of the NTD, but not other eIF4B domains also confers a dominant-negative overexpression phenotype in cells harboring temperature-sensitive mutant eIF4F alleles, repressing growth even under permissive temperature (Zhou et al., 2014). This suggests the NTD is needed to activate eIF4F. Deletion of either the NTD or 7-repeats domain also increases the amount of eIF4A needed to achieve maximal rate of mRNA recruitment to the PIC, suggesting both domains are needed for optimal functional interaction of eIF4A with the preinitiation complex (Walker et al., 2013). Evidence was recently provided for RNA-dependent interaction of the 7-repeats domain with eIF4A *in vitro* (Andreou et al., 2017), and for an RNA-independent interaction of eIF4B with eIF4F *in vitro* and *in vivo* (Park et al., 2013). These observations together suggest a model in which the seven repeats domain of eIF4B binds the preinitiation complex, where the NTD can effectively promote eIF4A activity or a conformation of the PIC that allows effective loading and scanning of mRNAs.

In contrast to the 40S binding activities of the NTD and the seven repeats domains, disrupting the RNA-binding activity of the RRM of yeast eIF4B did not affect growth or translation activity *in vitro* or *in vivo*, unless combined with other mutations that affect function on their own, such as deletion of the NTD or 7-repeats (Walker et al., 2013). This suggests that the RNA-binding RRM can stimulate the required function of the NTD and seven repeats, although to a limited extent which does not accelerate growth rate under standard laboratory conditions. The RRM is the only large functional domain with considerable sequence conservation between yeast and human eIF4B (Altmann et al., 1995) outside of small motifs of homology in the NTD and a segment of homology to the core sequence of a single yeast repeat just downstream of the RRM in human eIF4B. These motifs in the human factor are sufficient to rescue function of yeast eIF4B variants lacking the analogous motifs, suggesting they may provide a conserved function (Zhou et al., 2014).

Here we investigated the ability of the RNA-binding RRM and the 40S-binding NTD of eIF4B to stimulate translation and growth under various stress conditions. We found that the NTD enhanced growth in conditions that require robust cellular integrity, including in the presence of 3% w/v urea. Deletion of the NTD resulted in reduced eIF4B association with ribosome fractions and large decreases in translation as expected based on our previous work. Analysis of the structural content of mRNAs that strongly depended on the NTD for translation supports the model that eIF4B is necessary to enhance translation of mRNAs with long, structured 5′UTRs that showed less enrichment with eIF4G and other closed-loop factors, and further implicates the NTD in promoting this function (Sen et al., 2016). As expected, eIF4B interaction with translating polysomes was disrupted by truncation of the NTD, in agreement with our previous claim that this domain stabilizes binding to free 40S subunits (Walker et al., 2013). The mRNAs that responded to eIF4B NTD-deletion encode cell wall, membrane and ER/Golgi-associated proteins. Further analysis of the full complement of mRNAs in gene ontology classes associated with high NTD-dependence showed that mRNAs encoding membrane and trafficking proteins, irrespective of strong NTD-dependent changes in this study, had more structured 5′UTRs than other yeast mRNAs. The mRNAs that showed relative increases when the NTD was deleted were in contrast associated with cytoplasmic proteins, and especially with cytoplasmic translation. The mRNAs in these NTD-independent gene ontology classes showed significantly less structure. The divergence in mRNA structure propensity and likewise, eIF4B-dependence, of cytoplasmic proteins versus membrane-associated factors may allow the cellular responses to various stressors garnered by the eIF4B NTD. Together these data suggest eIF4B-NTD activity is needed to reprogram translation and allow cells to adapt to diverse cellular environments.

## Materials and Methods

### Construction of Yeast Strain and Plasmids

**FIGURE 2:**
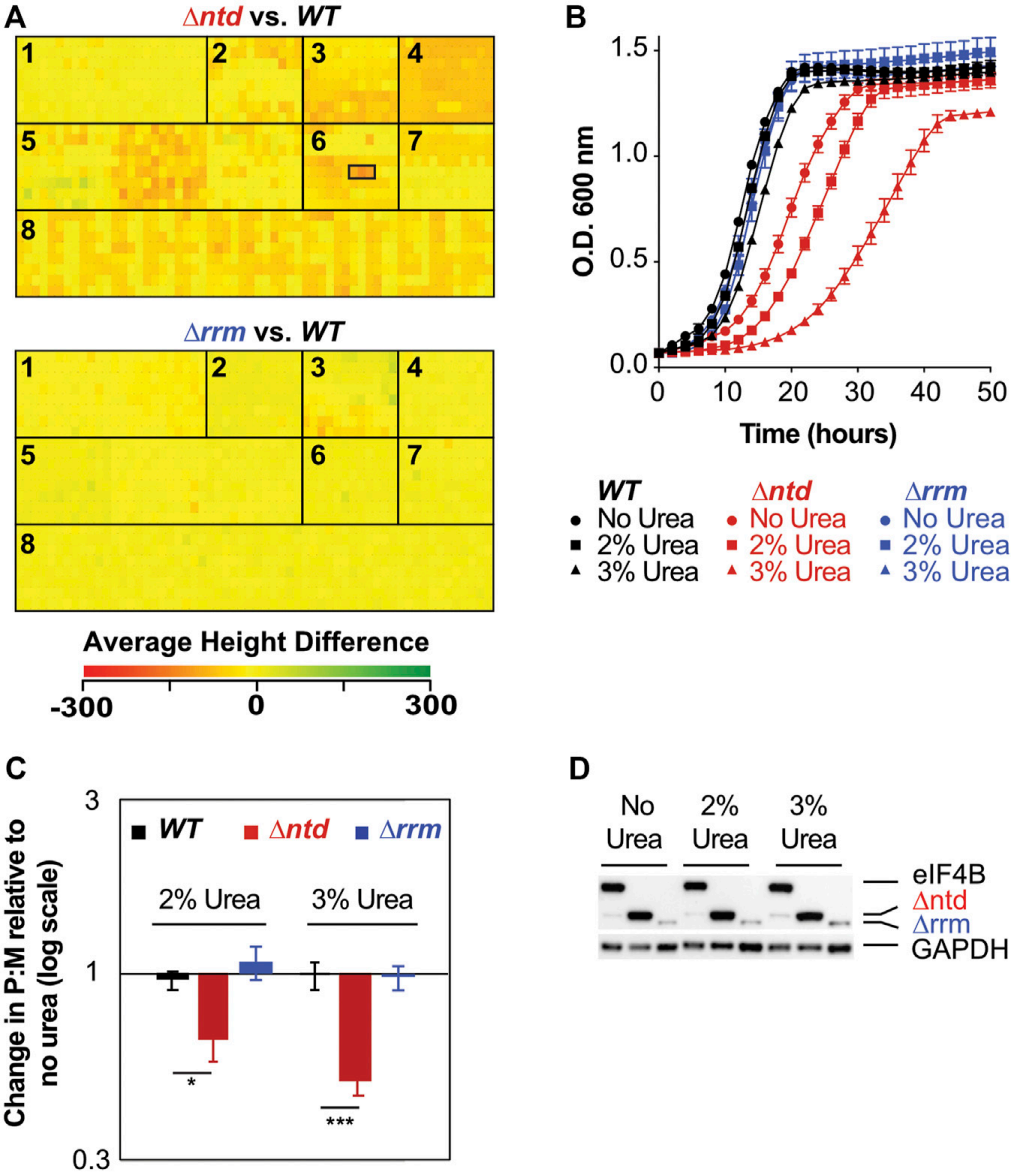
eIF4B binding to the 40S promotes resistance to stressors that challenge cellular integrity through changes in translation. **(A)** Heatmap of average height differences for cellular fitness between the NTD **(top)** or RRM **(bottom)** deletion mutant (green) and WT (red) under 1440 metabolic and chemical conditions as assayed by Phenotype microarray, which includes seven panels in 15 96-well plates: 1. Carbon Sources; 2. Nitrogen Sources; 3. Phosphorus and Sulfur Sources; 4. Nutrient Supplements; 5. Peptide Nitrogen Sources; 6. Osmolytes; 7. pH; and 8. Chemical Sensitivity. Wells containing 2, 3, and 4% urea are outlined by a black box, left to right, for ∆ntd. 3% Urea gave the largest difference in WT and *∆ntd* fitness. There were no conditions that gave a significant change for ∆rrm **(B)**. Growth curves of WT (black), ∆ntd (red), and ∆rrm (blue) grown with 0% (circles), 2% (squares), or 3% urea (triangles). Results from biological triplicates ± SEM are shown. **(C)** Relative change in polysome to monosome (P:M) ratio upon exposure to urea was determined as in 1B with representative traces shown in Supplementary Figure S2 in the presence and absence of 3% urea. Biological triplicates ± SEM are shown with *p* values from Student’s t test indicated (*p < 0.05). **(D)** eIF4B levels in WT, ∆ntd, and ∆rrm strains after growth with 0, 2%, or 3% urea in Synthetic Dextrose media.

Yeast strains (Supplementary Table S1) YSW3 and YSW4 were generated by tetrad dissection of strain FJZ001 (*MATa/MATα, his3Δ1/his3Δ1 leu2Δ0/leu2Δ0 met15Δ0/MET15 LYS2/lys2Δ0 ura3Δ0/ura3Δ0 TIF3/ tif3Δ::hisG-URA3-hisG*) (Walker et al., 2013), followed by isolation of LYS+, HIS−, LEU−, and MET− haploid clones, and finally counterselection on 5-FOA for removal of the *URA3-HisG* cassette of the URA+ *tif3∆* strain. *TIF3* alleles with the native promoter and terminator encoding C-terminal hexahistidine tagged-eIF4B or variants were Gibson-assembled (NEB, United States) into the BamHI site of single-copy vector pHLUM (Mülleder et al., 2012). DNA sequences of the entire PCR-amplified regions were verified in the assembled plasmids (Supplementary Table S2). Plasmids were transformed into YSW4 to generate prototrophic strains. Transformed strains were verified by Western blot for correct eIF4B variant expression (Figure 2D and Supplementary Table S1).

Plasmids (Supplementary Table S2) for monitoring translation reporter expression were constructed using the Mo-Clo yeast toolkit Golden Gate cloning system as described (Lee et al., 2015). The promoter, 5′UTR, and first 30 nucleotides of FIG2 and VBA2 genes were amplified from yeast (BY4741) genomic DNA and cloned into parts vector pYTK001 *via* BsaI assembly. An intermediate vector (pAS45) containing an *E. coli* GFP cassette (pYTK047) for screening green/white colonies by replacement of GFP was combined with the FIG2 and VBA2 parts vectors, the C-terminal Venus fusion tag (pYTK045), and a terminator part vector (pYTK053) for BsmBI assembly of the complete reporter vectors. Plasmids were verified by sequencing.

### Phenotype Microarrays

Previous work showed that inclusion on the same plasmid of the four requisite metabolic genes lacking in the parental genome of the barcoded yeast knockout collection (*HIS3*, *LEU2*, *URA3*, and *MET17*) provided a selective growth advantage, regardless of whether the metabolites these genes produce were included in the media. Hence, the plasmid with four metabolic markers is effectively retained even in rich growth media without selection, and synthetic growth effects between metabolic gene deletion and the mutation of interest can be avoided (Mülleder et al., 2012). Prototrophic strains YSW5, 6, and 7 were sent to Biolog for Phenotype Microarray screening at 30°C in duplicate, using plates 1–10 and 20–25 (Biolog Inc, United States) (Bochner et al., 2001). Plates were read every 30 min for 48 h.

### Yeast Growth

Yeast were cultivated in liquid or on solid (2% agar) Synthetic Drop-out (SD) media (20 g/L glucose, 1.71 g/L yeast nitrogen base without amino acids containing 5 g/L ammonium sulfate; Sunrise Scientific Products, United States) at 30°C. For assays with additives, yeast cells from an overnight culture in SD media were diluted to an OD_600_ of 0.05 in SD media or SD media supplemented with additive (e.g. 2% or 3% urea), and allowed to grow to mid-log phase with shaking at 30°C, unless otherwise noted. Automated growth curves were performed in 96-well plates (200 µl SD and additives per well) by taking OD_600_ measurements every 2 h for 48 h while incubating at 30°C with double-orbital shaking in a Spark plate reader (Tecan, Switzerland). The same trends in growth rate were observed on solid media (data not shown).

### Analysis of Polysome:Monosome Ratios

Polysome analysis was performed as described previously (Lee et al., 2007; Walker et al., 2013). For Figure 1B, yeast cells were cultured in SD medium with or without additives as noted at 30°C to an OD_600_ of 1.5. Cycloheximide (Gold biotechnology, United States) was added to a final concentration of 50 µg/ml and incubated for 5 min at 30°C with shaking before collecting cells by pelleting in centrifuge bottles packed with ice. Pellets were resuspended in 1/3 of the pellet weight of breaking buffer (20 mM Tris-HCl at pH 7.5, 50 mM KCl, 10 mM MgCl2, 1 mM DTT, 200 μg/ml heparin, 50 μg/ml cycloheximide, and one Complete EDTA-free Protease Inhibitor Tablet [Roche]/50 ml buffer), dropped into liquid nitrogen, and lysed in a Nitrogen Mill for 10 Cycles following a precool of 15 min with the following parameters: 1 min run, 2 min cool, rate = 15 (Spex Sample Prep, United States). 25 A_260_ units of lysates were resuspended in 1.5 volumes of the pellet weight of ice-cold breaking buffer and clarified by spinning at 14,000 rpm for 15 min at 4°C prior to separation by velocity sedimentation on 5–45% sucrose gradients (20 mM Tris-HCl [pH 7.5], 50 mM KCl, 10 mM MgCl_2_, 1 mM DTT, 5–45% sucrose mixed using the 5–45% SW-41 gradient program on the BioComp gradient station) by centrifugation at 39,000 rpm for 2.5 h at 4°C in a Beckman SW41 rotor. Gradient fractions were separated on a gradient station (BioComp, Canada) while scanning at 254 nm. The areas under the monosome and polysome peaks, determined in GraphPad Prism software, were used to calculate the P/M ratio.

### Analysis of Initiation Factor Association With Subunits and Polysomes by Crosslinking and Gradient Ultracentrifugation

To monitor association of eIF4B and other proteins with 40S subunits, formaldehyde crosslinking analysis was performed as described (Herrmannová et al., 2020) with the following differences: Yeast cells were grown to an OD_600_ of ∼0.8 in SD media at 30°C. Cultures were poured into bottles packed with ice containing formaldehyde for a final concentration of 2% and incubated for 60 min prior to collection and lysis. Cells were lysed in a nitrogen mill as described above and resuspended to 20 A_260_ units per 300 µl. 300 µl of crosslinked lysates were then loaded and separated by spinning at 41,000 rpms for 5 h on 7.5–30% sucrose gradients in an SW-41 rotor, and 0.63 ml fractions were collected upon fractionation on a Biocomp gradient station. Fractions 1–2 were combined prior to loading. Fractions up to the 40S peak were analyzed by western analysis. Three biological replicates were performed.

For observing polysome association, Cycloheximide was added to the culture to a final concentration of 50 µg/ml and incubated for 5 min at 30°C with shaking before harvesting cells on ice. Cells were lysed and resuspended in BBK buffer as above, then formaldehyde was added to the resuspended lysates at a final concentration of 1%. Crosslinking was carried out for 30 min on ice before quenching with glycine at 0.1 M. Crosslinked cell lysates were layered on 5–45% gradients and 10 1 ml fractions were collected for analysis by western blotting (Herrmannová et al., 2020). Two biological replicates were performed.

### Western Analysis

Antibodies and samples were used at concentrations that showed linear increases when samples were titrated. TGS and TGX-Stain-free gels were transferred to PVDF membranes using the Trans-blot Turbo system (Biorad, United States). Visualization of blots was performed using BioRad ECL or ECL Max sensitivity HRP substrate as needed for the secondary antibody. When blotting the same protein on multiple blots for comparison, the acquisition time was kept approximately the same. The stain-free visualization of total protein was carried out using a Biorad touch imager according to the manufacturer’s protocol.

To verify that eIF4B variants were expressed under stress conditions, yeast cells were grown in SD media with or without 2 or 3% urea, and harvested at an OD_600_ of 1.0. Whole cell extracts (WCEs) were prepared by extraction with trichloroacetic acid (TCA) and subjected to Western blot analysis as described previously (Walker et al., 2013) using antibody against the His6 epitope (EMD Millipore/Novagen 70796, 1:2000 dilution) Experiments were repeated three or more times from biological replicates.

For analysis of eIF4B position within gradients, 0.5 ml of each gradient fraction was precipitated by addition of 1 ml 100% Ethanol and spinning for 30 min at 13,000 × g, resuspended in SDS loading buffer, and resolved by SDS-PAGE, followed by Western blotting using anti-His antibody. 40S subunit (and 80S/polysome) containing fractions were verified from the same samples by blotting yeast ribosomal small subunit protein Rps2 (Aviva ARP63572_P050, 1:2000 dilution.) Rabbit antibodies to purified recombinant yeast eIF4A (1:20,000 dilution) and eIF4G1 (1:1000 dilution), generated by Invitrogen/Pierce custom antibody services and verified against the recombinant proteins, were used to determine the position of those proteins within gradients (Liu et al., 2019). Each experiment was repeated three or more times from biological replicates.

To determine changes in *FIG2* and *VBA2* translation reporter fusions, TCA-precipitated lysates were prepared as above from cells grown in media with or without 3% urea to an OD_600_ of 0.5–0.8. Lysates were separated on a stain-free SDS-PAGE gel (Bio-rad), then blotted with mouse anti-GFP antibody (Roche 11814460001, 1:1000 dilution) and anti-mouse-HRP secondary (Cell Signaling 7076, 1:3500). GFP was normalized to total protein bands per lane (visualized by a stain-free scan on a Bio-rad gel doc), which was also normalized prior to loading lysates. Each experiment was repeated at least two times from biological replicates.

### RiboSeq and RNASeq Library Preparation

Ribosome footprint profiling was conducted as described (Ingolia et al., 2009; Guydosh and Green, 2014; McGlincy and Ingolia, 2017) with minor modifications. Yeast cells at an OD_600_ of approximately 0.8 were rapidly harvested by vacuum filtration through a 0.45 µm Whatman cellulose nitrate membrane filter (GE Healthcare Life Sciences, United States) at room temperature by scraping the slurry into liquid nitrogen. Cells were lysed as above in a Nitrogen mill, and thawed and suspended in lysis buffer (20 mM Tris [pH 8], 140 mM KCl, 1.5 mM MgCl_2_, 1% Triton X-100, 100 µg/ml cycloheximide). 25 A_260_ units of extract were treated with 87.5 Units of RNase If (M0243, NEB, United States) for 1 h at 22°C on a rotator, then separated on 10–50% sucrose gradients (20 mM Tris [pH 8], 140 mM KCl, 1.5 mM MgCl_2_, 1 mM DTT, 100 µg/ml cycloheximide, 10–50% sucrose) by centrifugation at 40,000 rpm for 3 h at 4°C in a Beckman SW41 rotor and fractionated as above. Ribosome-protected RNA footprints were purified from the nuclease-treated monosome fraction by addition of SDS to 0.8% at 65°C, followed by extraction with acid phenol [pH 4.5] (Ambion, United States) and then chloroform/isoamyl alcohol extraction. 300 mM NaOAc [pH 5.2] was added to the aqueous phase and RNA was precipitated with one volume of isopropanol before resuspending in 10 mM Tris-HCl [pH 8]. RNA footprints from 25 to 35 nt were size-selected on a 15% denaturing PAGE gel, eluted by crushing and soaking gel fragments in RNA elution buffer (300 mM NaOAc [pH 5.5], 1 mM EDTA, 0.1 U/µl SUPERaseIn (Life Technologies, United States)), and dephosphorylated using T4 Polynucleotide Kinase (M0201, NEB, United States) prior to isopropanol precipitation and resuspension in 10 mM Tris [pH8]). A pre-adenylated universal linker (5′-rAppCTGTAGGCACCATCAAT-NH_2_-3′) was prepared in house or purchased from NEB (S1315S) and ligated to the 3′ ends of the dephosphorylated footprints using T4 RNA Ligase 2, truncated (MO242L, NEB). rRNA was depleted using the Yeast Ribo-Zero Gold rRNA removal kit (Illumina, United States). First strand synthesis was performed with Superscript III (Life Technologies, United States) and reverse transcription primer NINI9 (5’-/5Phos/ AGA TCG GAA GAG CGT CGT GTA G GGA AAG AGT GTA GAT CTC GGT GGT CGC/SpacerC18/ CAC TCA/SpacerC18/ TTC AGA CGT GTG CTC TTC CGA TCT ATT GAT GGT GCC TAC AG), followed by circularization with Circligase (Epicenter, United States). Circularized cDNA was then PCR amplified using primer NINI2 (AAT GAT ACG GCG ACC ACC GAG ATC TAC AC) and a primer with a barcode (CAA GCA GAA GAC GGC ATA CGA GAT XXX XXX GTG ACT GGA GTT CAG ACG TGT GCT CTT CCG), where XXXXXX denotes a six-nucleotide-barcode used to distinguish samples run in the same lane (Supplementary Table S4). For RNA-seq, total RNA was isolated from the same cell extracts using SDS/hot acid phenol/chloroform extraction. The Ribo-Zero Gold Yeast kit was used to remove rRNA, and total RNA was randomly fragmented by incubating for 20 min at 95°C in freshly made fragmentation buffer (100 mM sodium carbonate-bicarbonate [pH 9.2], 2 mM EDTA). RNA was then precipitated and fragments of 40–60 nt were purified from a denaturing PAGE gel, and library generation carried out as above. Ribo-Seq and RNA-Seq were performed for two independent cultures for each condition (WT and ∆ntd cells grown in SD both with and without 3% Urea), and the 16 libraries sequenced in two lanes with 150 bp reads on an Illumina HiSeq 4000 instrument by Genewiz.

### Analysis of Ribosome Profiling Libraries

The Ribogalaxy platform (https://ribogalaxy.ucc.ie, (Michel et al., 2016)) was used for trimming linker sequences (Cutadapt version 1.1.a; (Martin, 2011)), subtractive alignment of *S. cerevisiae* non-coding RNAs (Bowtie version 1.1.a; (Langmead et al., 2009); using the R64.2.1 S288C genome from Saccharomyces Genome Database (SGD, RefSeq ID: 285498), alignment of rRNA subtracted libraries to the transcriptome (Bowtie version 0.1.3 using the SGD transcriptome dataset and counting of uniquely mapped reads (Ingolia et al., 2009) using Ribocount version 0.3.1. Statistical analyses of differences in total RNA counts, ribosome footprints, or TE values between WT, mutant, urea-treated, and untreated samples were conducted using DESeq2 and are presented in Supplementary Table S6 along with the calculated false discovery rates (Love et al., 2014). Gene ontology categorization of library-specific differences was performed at SGD, using all genes within the four classes, NTD dependent increase/decrease in urea, NTD dependent increase/decrease in SD (Thompson et al., 2016). Cumulative PARS scores (Kertesz et al., 2010) and strong-closed loop association (Costello et al., 2015) for yeast mRNAs were obtained from published works and were analyzed for those mRNAs showing reduced or enhanced dependence on the NTD with and without urea, as described previously for cells lacking eIF4B (Sen et al., 2016).

Gene set enrichment analysis (GSEA) was carried out by using the curated gene sets of Gene Ontology for *S. cerevisiae* (http://ge-lab.org/gskb/), (Supplementary Figure S5) The list of the entire detectable genes with log_2_ ratios derived from each comparison was used for the pre-rank GSEA, and we followed the standard procedure described by GSEA user guide (http://www.broadinstitute.org/gsea/doc/GSEAUserGuideFrame.html). The nominal *p*-value is the statistical significance of the enrichment score.

### qRT-PCR

Cell lysates were prepared and 12 fractions were collected from polysomes fractionated using the protocol for Riboseq gradient preparation above (without nuclease treatment.) 0.3 ml of each gradient fraction was spiked with equal amounts of control RNA (Fluc mRNA, Trilink Biotechnologies, United States), then total RNA was extracted using the hot acid phenol-chloroform method (Thompson et al., 2016). First strand synthesis was performed with iScript Advanced Reverse Transcriptase (Biorad, United States) using oligo-dT primers and random hexamer primers. Quantitative PCR was performed with iQ SYBR Supermix reagents (Biorad, United States) using CFX384 Touch Real-Time PCR detection system (Biorad, United States) two times per sample. Gene-specific primer sequences are listed in Supplementary Table S3.

## Results

Previous work demonstrated that the N-terminal domain (NTD) of eIF4B promotes both affinity for the 40S subunit *in vitro* and recruitment of mRNAs to the preinitiation complex *in vitro* and *in vivo*, while the RRM of eIF4B is dispensable in auxotrophic yeast strains (Walker et al., 2013; Zhou et al., 2014). Given the diverse dependencies of cellular mRNAs on eIF4B function (Sen et al., 2016), we thought it likely that differential mRNA selection promoted by eIF4B could confer phenotypic advantages. We wondered if the ability of the NTD to promote translation would afford cells the ability to resist different stressors, and whether the RRM could provide additional function under stress, so we constructed prototrophic strains for phenotype microarray analysis. We previously reported that deletion of the NTD conferred slow growth and cold-sensitivity in a strain auxotrophic for histidine, uracil, and methionine on solid media (Coppolecchia et al., 1993; Walker et al., 2013). For this work, an eIF4B null mutant was transformed with a plasmid that complemented all four existing auxotrophic markers and provided a WT, ∆ntd, or ∆rrm eIF4B gene copy (*TIF3, tif3 ∆ntd,* or *tif3 ∆rrm*) under the native promoter and terminator (Figure 1A). As previously reported, deletion of the NTD, but not the RRM reduced growth rate (Figure 2B, (Walker et al., 2013)).

Polysome profiles from these prototrophic strains expressing WT, ∆ntd, and ∆rrm eIF4B confirmed that NTD deletion led to a gross reduction of polysomes and an increase in monosomes in the mutant (Figure 1B and Supplementary Figure S2), indicating the NTD promotes global translation initiation *in vivo* in a prototrophic background. RRM deletion had only a minor effect on polysome to monosome ratio when expressed from this single copy plasmid (Figure 1B, blue), in agreement with our previous findings (Walker et al., 2013).

### The NTD of Yeast eIF4B Enhances Association With Ribosome Complexes *In Vivo*

We previously reported that deletion of the NTD decreased binding affinity of eIF4B for purified 40S subunits. In addition, deletion of the NTD decreased the rate constants and endpoints of mRNA binding to the PIC *in vitro*, while affecting the conformation of two areas of the rRNA near protein RPS20/uS10 (Walker et al., 2013). To determine whether deletion of the NTD affected association of eIF4B with ribosomes in yeast, we performed velocity gradient fractionation of formaldehyde-crosslinked lysates followed by Western blotting of eIF4B, eIF4A, eIF4G, and Rps2. We performed two types of gradients to observe changes in association of eIF4B with both translating ribosome complexes and 40S subunits and PICs as a function of NTD and RRM deletion (Figures 1C–I and Supplementary Figure S1). Running crosslinked lysates on a 5–45% gradient effectively separates polysome-, monosome-, and mRNP-fractions. We then blotted for eIF4B in each fraction of the gradient and determined distribution of eIF4B within each fraction of the gradient. Importantly, we found that ∼55% of WT eIF4B comigrated with Rps2/40S subunit-containing fractions (Figures 1C,D fractions 4–10, Figure 1H), both as part of the 40S/PIC and moreso with the later fractions containing translating polysome complexes, which make up more of the ribosome pool. Upon deletion of the NTD we saw that Rps2 shifted from later to earlier fractions, confirming the polysome to monosome shift observed by UV spectroscopy in this paper (Compare Figures 1B,D) and in prior work (Walker et al., 2013). This indicates deletion of the NTD led to less ribosomes associated with mRNAs, suggesting reduced translation initiation rate in these cells, as we previously reported. In addition, we found that upon deletion of the NTD, eIF4B position in the gradient was shifted such that ∼80% of the protein moved to the first two fractions that lack 40S subunits and ribosomes (Figure 1C, compare black and red.) This is in contrast to deletion of the RRM, which conferred only a minor decrease in translating ribosome capacity, judged by similar polysome:monosome ratio as WT (Figure 1B, compare black and blue), and also did not grossly affect eIF4B association with translating ribosomes (Figures 1C,D).

Ultracentrifugation of crosslinked lysates on 7.5–30% sucrose gradients optimally separates 40S/PIC fractions (Supplementary Figure S1) We found that WT eIF4B was present in fractions 10–13 that contain RPS2 and indicate 40S, 43S, and 48S complexes. As we saw in Figure 1, eIF4B was also present in early fractions containing mRNPs (Supplementary Figures S1A,C). Deletion of the NTD reduced the amount of eIF4B in these Rps2-containing fractions by 93%, with increased eIF4B in early fractions containing proteins and mRNPs.

We also found that while the RRM did not change the amount of eIF4B comigrating with the overall ribosome pool (Figure 1), deletion of this domain did decrease occupancy of eIF4B on 40S/PICs, although not to the extent observed for NTD deletion, which nearly eliminated eIF4B occupancy in PIC fractions (Supplementary Figure S1). It is notable that deletion of the RRM decreased the concentration of eIF4B in cells (Figure 2D), and was previously shown to have a minor effect on 40S binding affinity and apparent affinity for the PIC in an mRNA recruitment assay (Walker et al., 2013), so this decrease in RRM occupancy of PICs outside of polysomes could be a reflection of that decreased ribosome affinity.

As a control we blotted the same gradient fractions for additional 48S components, eIF4A and/or eIF4G (Figures 1E,F and Supplementary Figure S1B). In contrast to eIF4B, we found that eIF4A and eIF4G remained distributed across gradient fractions when the NTD or RRM of eIF4B were deleted. This suggests the affinity of eIF4A and eIF4G for the PIC are not mediated by eIF4B. Moreover, this suggests that shift of ∆ntd eIF4B from polysome and 40S fractions to ribosome-free fractions is the result of decreased ribosome binding affinity of the mutant, and less likely to be due to eIF4F not associating with mRNPs as a result of eIF4B inactivation, since these other components of 48S PICs did not show a PIC and polysome to mRNP shift.

### Deletion of the NTD of eIF4B Results in Decreased Resistance to Stressors and Associated Decreases in Translation

To determine conditions under which the NTD of eIF4B, which promoted association with ribosomes, plays a specific role in regulating growth and translation, we performed phenotype microarrays of cells expressing WT or ∆ntd eIF4B from a single copy plasmid that also restored nutrient prototrophy. As an additional control, we performed phenotype microarray analysis on cells expressing ∆rrm eIF4B. Phenotype microarray analysis was not performed on cells expressing eIF4B lacking the seven repeats or a strain lacking eIF4B entirely, because the growth rates of these strains are too slow under optimal growth conditions to clearly assess additional effects of stressors in these assays. Phenotype microarray analysis showed a large number of conditions in which the prototrophic ∆ntd eIF4B-expressing strain grew at a reduced rate compared to the wild-type eIF4B expressing cells (Figure 2A). The strongest responses (Supplementary Table S5) include osmolytes, detergents, a number of peptides as nitrogen sources, and antibiotics. Urea, which gave the strongest negative phenotype when present at 3% w/v in the media, acts as a denaturing agent, can cause membrane blebbing at high concentrations (Lambert and Draper, 2012; Necas and Svoboda, 1973), and can readily cross the yeast cell wall and membrane to act as a nitrogen source (Cooper and Sumrada, 1975). Tamoxifen, which targets the estrogen receptor in higher eukaryotes, targets the calmodulin protein in yeast, which regulates stress responses through Hog1 interaction (Dolan et al., 2009; Kim et al., 2016). Poly-L-Lysine can act as a cationic detergent or a charged adherent for various molecules, and likely interacts with the cell wall or membrane. The strain lacking the NTD of eIF4B also showed heightened sensitivity to antibiotics that target the small ribosomal subunit, apramycin sulfate and to a lesser extent tobramycin. WT yeast are not sensitive to these antibiotics, which when combined with the sensitivity to various salts (Potassium chloride, Chromium chloride, and to a lesser extent, sodium chloride) and other phenotypes described above, suggests a defect in membrane and/or cell wall permeability in the mutant. Growth defects were verified for ∆ntd-expressing cells in the presence of urea, which conferred the largest reduction in the mutant (Figure 2B, red). These experiments confirmed that WT growth rate is nearly unaffected by urea (Figure 2B, black) while the mutant shows slow growth. In contrast, deletion of the RNA-binding RRM domain, which diminishes *in vitro* RNA-binding affinity (Walker et al., 2013), conferred no large reproducible advantage or hindrance in the phenotype microarray, and supported levels of growth similar to WT eIF4B, suggesting the RNA-binding activity of eIF4B, at least that provided by the RRM, is dispensable for growth in all conditions tested (Figure 2B, blue). Together these results suggest the eIF4B 40S-binding NTD is required for resistance to a number of growth conditions that challenge cellular integrity, and that the mutant may have a defect in membrane and/or cell wall permeability.

To further investigate the mechanisms by which the NTD promoted growth in the presence of stressors, we compared polysome traces for WT and ∆ntd eIF4B-expressing cells grown in the presence and absence of 2% or 3% urea (Figure 2C, traces shown in Supplementary Figure S2). Whereas WT eIF4B-containing cells showed minor decreases in polysome to monosome (P:M) ratio upon addition of either concentration of urea (7% reduction in 2 and 10% reduction in 3% urea; Figure 2C, black), ∆ntd cells showed further 36 and 50% decreases in P:M ratio due to urea exposure (Figure 2C, red). In contrast, deletion of the RRM resulted in less than 5% change in P:M ratio (Figure 2C, blue).

The reduction in translation conferred by NTD deletion was not due to altered levels of eIF4B, as immunoblotting for a His tag on the eIF4BC-terminus showed similar protein levels when grown in media with varied urea concentrations (Figure 2D). As noted before (Walker et al., 2013), deletion of the RRM resulted in reduced detection of that protein. As previously stated (Walker et al., 2013), the reduction in ∆rrm protein levels without a large decrease in polysome:monosome levels or growth rate suggests eIF4B may normally be present in excess of what is required to stimulate translation and growth. While perplexing that the factor level can be reduced to such a large extent without corresponding functional defects, this is in agreement with the observation that targeted reduction of WT eIF4B levels by ∼55–75% resulted in only a minor reduction in translation rate in yeast (cells retained ∼80–95% of WT translation rate, (Firczuk et al., 2013).) However, the level of detectable ∆rrm protein, while lower than that of WT and ∆ntd eIF4B, was unaffected by urea addition, suggesting urea did not affect the expression or stability of the protein. Together, these data suggest interactions and activities promoted by the NTD within WT eIF4B allow robust translation in the presence of urea.

### Activities Supported by the NTD Promote Translation of mRNAs Encoding Membrane-Associated Proteins

**FIGURE 3:**
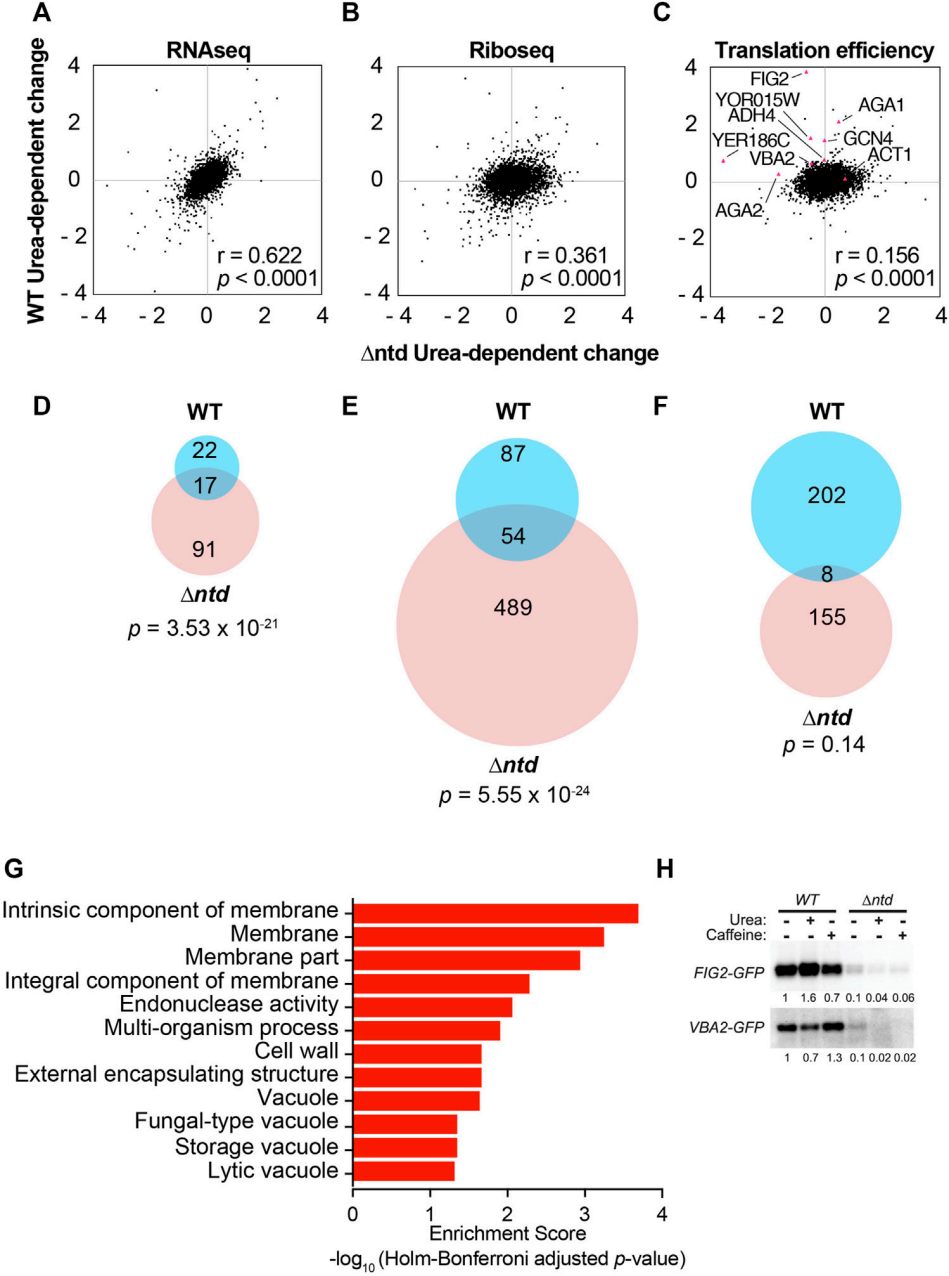
The NTD of eIF4B promotes translation of mRNAs encoding proteins associated with the membrane and cell wall. **(A**–**C)** Comparison of Log_2_ values for changes in RNAseq **(A)**, Riboseq **(B)**, or TE **(C**, Translation efficiency) in response to 3% urea for WT are plotted vs. the Log_2_ values for changes observed in the NTD deletion mutant for each of the 4070 genes with measurable expression in each group. Nine mRNAs are indicated in **(C)**, which were analyzed by qRT-PCR of polysome gradient fractions in Supplementary Figure S3 Pearson correlation coefficients are shown. **(D**–**F)** Overlap of urea-dependent genes exhibiting 1.5-fold or greater increase in RNAseq **(D)**, Riboseq **(E)**, or TE **(F)** for WT and *∆ntd*. The Fisher’s exact *p*-values were shown to indicate the statistical significance of overlap between two datasets. **(G)** Gene ontology analysis for urea-dependent RNAs (e.g. increased TE in WT in response to urea.) **(H)**. Western analysis of GFP translation reporters. The 5′UTR and first 30 nucleotides of the FIG2 and VBA2 genes were fused to GFP in a plasmid under the native promoters for each. Indicated transformants of WT and ∆ntd eIF4B-expressing yeast were subjected to anti-GFP western analysis following growth in the absence or presence of 3% urea or 1.5 mg/ml caffeine. The fractions of reporter band intensity per total protein bands for each lane on the gel were normalized to WT eIF4B without additive for each reporter.

We next performed ribosome profiling on the yeast cells expressing WT or ∆ntd eIF4B, both with and without urea to determine how eIF4B•40S association affects translation of individual mRNAs. Ribosome profiling maps the positions of translating ribosomes on mRNAs to determine which sequences are translated more and less effectively in response to changes. We prepared illumina-indexed cDNA libraries for RNAseq and Riboseq from WT and ∆ntd eIF4B-expressing cells in the presence or absence of 3% urea. Comparison of replicates indicates sufficient reproducibility for each sample, with slightly higher variability in the Riboseq libraries from ∆ntd eIF4B-expressing cells with 3% urea (Supplementary Figure S3). This sample showed the strongest global repression of translation by polysome:monosome assessment (Supplementary Figure S2) and therefore had the least ribosome footprints, so the increased noise is expected. After mapping and quantifying footprints on RNAs in the yeast transcriptome, we compared the log_2_fold-changes in RNAseq, Riboseq, and TE (translation efficiency, the ratio of Riboseq/RNAseq) in response to urea exposure in WT versus the urea-dependent change (log_2_fold change in TE for the same strain with and without urea) in ∆ntd eIF4B-expressing cells (Figure 3 and Supplementary Figure S3 and Supplementary Table S6). Changes in individual RNA levels in response to urea were more similar between the two strains (Figure 3A, *r* = 0.622), while changes in ribosome footprints and TE for individual RNAs in response to urea showed less correlation between the two strains (Figure 3B, Riboseq, *r* = 0.361; 3C, TE, *r* = 0.156). This is further evidenced by evaluation of the RNAs with ≥1.5-fold increase in RNAseq, Riboseq, and TE in response to urea. There were fewer RNAs overall showing ≥1.5-fold urea-dependent increases in RNA levels (Figure 3D; WT = 39, ∆ntd = 108). In contrast, there were large numbers of RNAs with ≥1.5-fold changes in Riboseq (Figure 3E, WT = 141 RNAs, *∆*ntd = 543 RNAs) and TE in response to urea (Figure 3F, WT = 210 RNAs, ∆ntd = 163 RNAs), both in the WT and ∆ntd eIF4B-expressing cells. The pools of RNAs showing increased TE upon exposure to urea were almost completely distinct in control cells versus those RNAs whose TE increased in the ∆ntd mutant (3.8% of the WT TE change was also observed in ∆ntd, which represented 4.9% of the ∆ntd change; *p* value for overlap = 0.13). This is in contrast to the urea-dependent changes observed for RNA and Riboseq, in which the normal WT response to urea was represented significantly in the pools of RNAs also showing changes in the mutant (RNASeq, 44% of WT RNA change occurred in ∆ntd, *p* = 3.54 × 10^−21^; Riboseq, 38% of the WT Riboseq change occurred in ∆ntd, *p* = 5.55 × 10^–24^.) These overlapping RNAs constituted considerably less of the RNA and riboseq changes in ∆ntd (RNASeq, 16% of ∆ntd change; Riboseq, 9.9% of ∆ntd change overlaps with WT change). It is important to note that while there are more RNAs showing a urea-dependent increase in ribosome footprint density in ∆ntd than WT, the ∆ntd cells show an overall decrease in global translation initiation capacity as evidenced by decreased P:M ratio (Figures 1B, 2C). Together these data suggest that the mutation primarily prevents increased ribosome loading of specific mRNAs in urea, but that there are also dysregulated changes in RNA levels in the mutant.

Changes in ribosome occupancy of nine selected RNAs were verified by performing qRT-PCR on RNA from polysome gradient fractions (Figure 1B and Supplementary Figure S4) of cells expressing WT and ∆ntd eIF4B in the presence and absence of 3% urea in the growth media. The trends observed by ribosome profiling were confirmed by this independent method for mRNAs and 18S rRNA (Supplementary Figure S4 and Supplementary Table S10). Moreover, to determine whether changes in ribosome footprint density correlated with changes in protein production, we designed two translation reporters in which the 5′ UTR and first 30 nucleotides of two RNAs that showed NTD-dependent reductions (*FIG2* and *VBA2*) were cloned in frame in front of a GFP gene (Supplementary Table S2). Western blotting for GFP in extracts of cells harboring both a *FIG2-GFP* reporter and WT or ∆ntd eIF4B showed that addition of urea to WT cultures increased the level of FIG2-GFP protein by 1.6-fold (relative to total protein quantified per lane on gel after loading a normalized amount of lysate in each lane; Figure 3H). In contrast, addition of urea to WT cells did not increase steady state levels of VBA2-GFP, and instead resulted in a minor decrease (Figure 3H). This result is consistent with the increased polysome association observed for *FIG2* upon urea addition, but little to no change in polysome association observed for *VBA2* in WT cells upon urea addition (Supplementary Figure S4). In addition to monitoring the effects of urea, we determined the effect of caffeine on production of these reporters. Caffeine severely impaired growth of the ∆ntd mutant in the phenotype microarray assay (Figure 2A and Supplementary Table S5), so we reasoned that it may also affect reporter protein production if both conditions require function of eIF4B’s NTD. We found that caffeine incurred a modest decrease in *FIG2-GFP* production and a minor increase in *VBA2-GFP* production in the *WT* strain. In contrast, large decreases in translation were observed upon addition of caffeine in the *∆ntd* mutant, leading to little production of either reporter, even though *WT* and *∆ntd* cells were grown to the same degree prior to the western. Importantly, deletion of the NTD reduced the steady state levels of both reporter proteins by 90% or more, supporting a claim that reduced translation initiation upon deletion of the NTD may lead to reduced levels of some proteins that are highly reliant on eIF4B in order to be synthesized. GFP production in both strains was also verified by following fluorescence, and accumulated at a reduced rate in ∆ntd cells in the presence of urea (data not shown). Together these data suggest that the eIF4B•40S binding NTD may confer changes in growth by differentially affecting translation of specific mRNAs in response to stressors, although further analyses of proteome changes are needed to support that claim.

To understand how eIF4B NTD-dependent TE changes relate to enhanced growth in urea, we first performed gene ontology (GO) analysis of the mRNAs showing ≥1.5-fold increased TE in response to urea in WT or mutant cells (Figure 3G and Supplementary Figure S5). Of the 202 mRNAs with ≥1.5-fold increased translation in WT cells upon exposure to urea, 102 mRNAs were associated with the parental membrane (GO) term. Significant numbers of mRNAs were also associated with the cell wall, cellular periphery, and other related terms, suggesting the eIF4B NTD enhanced translation of mRNAs encoding proteins that remodel or otherwise localize to the cellular membrane. In contrast, of 155 mRNAs showing ≥1.5-fold increased translation in ∆ntd-expressing cells in response to urea, 151 of those mRNAs were associated with the cytoplasm GO term (Supplementary Table S7). Likewise, analysis of the mRNAs showing increased TE in ∆ntd cells showed strong association with GO terms for ribosomes and cytosolic components, even without urea (Supplementary Table S8). Furthermore, mRNAs that showed decreased TE in *∆ntd* cells in the absence of urea were associated with membrane-bound organelles (Supplementary Table S9). This suggests the NTD promotes translation of mRNAs encoding membrane-associated proteins, and the loss of this ability results in the mutant translating mRNAs that encode cytoplasmic proteins, leading to urea and other stress sensitivities.

Finally, we analysed the 281 mRNAs showing more than 1.5-fold decreased translation efficiency in response to urea in the mutant cells relative to WT. In this case we saw decreased translation for 84 mRNAs associated with the endomembrane system (*p*-value = 0.000026), including association with the ER, Golgi, transferase activities, glycosylation, and mannosylation (Table 1). This suggests effective translation of mRNAs for proteins trafficked through the ER/Golgi network to the membrane and cell wall may be dependent on eIF4B NTD activities. Together these results suggest that the eIF4B 40S-binding NTD may affect critical changes in translation of RNAs that remodel the cellular periphery in response to urea exposure. This function is necessary for translation of the optimal pool of mRNAs to promote rapid growth in standard media as well.

**TABLE 1:** Gene Ontology analysis for genes with decreased translational efficiency in ∆*ntd* compared to WT in the presence of urea.

GO term	*p*-Value
Transferase activity, transferring hexosyl groups [GO:0016758]	1.62E−06
Endomembrane system [GO:0012505]	1.44E−05
Transferase activity, transferring glycosyl groups [GO:0016757]	2.22E−04
Protein glycosylation [GO:0006486]	3.76E−04
Macromolecule glycosylation [GO:0043413]	3.76E−04
Mannosylation [GO:0097502]	5.19E−04
Glycoprotein biosynthetic process [GO:0009101]	8.19E−04
Cellular bud [GO:0005933]	1.55E−03
Protein N-linked glycosylation [GO:0006487]	1.91E−03
Glycosylation [GO:0070085]	2.40E−03
Glycoprotein metabolic process [GO:0009100]	2.85E−03
Cell periphery [GO:0071944]	4.09E−03
Golgi cisterna [GO:0031985]	4.70E−03
Cell part [GO:0044464]	8.14E−03
Cell [GO:0005623]	8.81E−03
Protein O-linked glycosylation [GO:0006493]	1.98E−02
Golgi stack [GO:0005795]	2.26E−02
Mannosyltransferase activity [GO:0000030]	3.04E−02
Organelle subcompartment [GO:0031984]	3.65E−02
Membrane part [GO:0044425]	4.69E−02

### Deletion of the NTD of eIF4B Reduces Translation Efficiency of mRNAs With Long and Structured 5′UTRs

**FIGURE 4:**
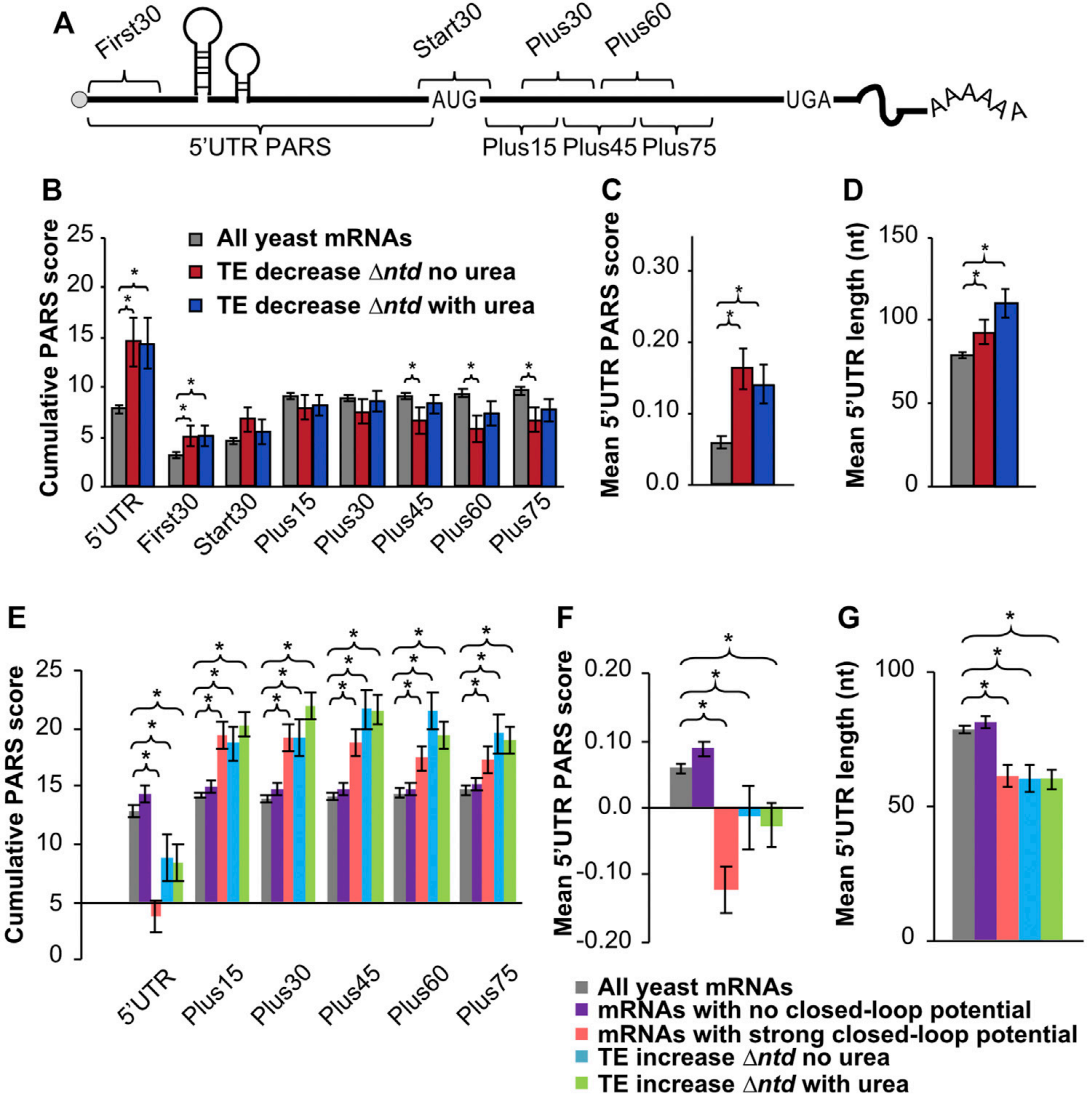
Comparison of PARS (Parallel analysis of RNA structure) scores indicates a higher propensity for secondary structure in the 5′UTRs of mRNAs that are dependent on the NTD for translation, and less associated with closed-loop factors. **(A)** Schematic showing 5′-UTR and CDS intervals for cumulative PARS scores. The sum of scores for all 5′-UTR nucleotides (5′UTR PARS); for the first 30 nucleotides (First 30 PARS); for 30 nucleotides surrounding the start codon (Start 30 PARS); and for nucleotides within the ORF, from +1 to +30 (Plus15), +16 to +45 (Plus30), +31 to +60 (Plus45), +46 to +75 (Plus60), and +61 to +90 (Plus75). **(B**,**C)** The mean PARS scores (calculated from data reported in reference (Kertesz et al., 2010)) for indicated cumulative regions **(B)**, or for individual nucleotides in the 5′UTR **(C)** are indicated for all yeast mRNAs with available PARS scores (gray, *n* = 2679); mRNAs with decreased TE (≥1.5-fold) in *∆ntd* relative to WT in media lacking urea (red, *n* = 138); and for mRNAs with decreased TE in *∆ntd* relative to WT in 3% urea (navy, *n* = 156). **(D)** Average length of 5′-UTR for the indicated sets of mRNAs. **(E**–**G)** PARS and 5′UTR length analysis calculated for the indicated gene sets, with *p* values from Student’s t test indicated (*p < 0.05): grey bar, all yeast mRNAs with available PARS scores (*n* = 2679); purple bar, mRNAs with no closed-loop potential, characterized for de-enrichment in immunoprecipitation of eIF4F and Pab1, and enrichment in immunoprecipitation of eIF4E-binding proteins as shown in (Costello et al., 2015); red bar, mRNAs with strong closed-loop potential, characterized for de-enrichment in immunoprecipitation of eIF4E-binding proteins and enrichment in immunoprecipitation of eIF4F and Pab1, as shown in (Costello et al., 2015); blue bar, mRNAs with increased TE in NTD deletion mutant as compared to WT without urea; green bar, mRNAs with increased TE in NTD deletion mutant as compared to WT in 3% urea. **(E)** Average PARS scores calculated for the indicated sets of mRNAs for each 5′-UTR or CDS interval described in Figure 4A. **(F)** Average PARS score calculated for entire 5′-UTR for the indicated sets of mRNAs. **(G)** Average length of 5′-UTR for the indicated sets of mRNAs.

Previous studies have shown that mammalian eIF4B is necessary for PIC assembly at the start codon of mRNAs with 5′UTR secondary structure *in vitro* (Dmitriev et al., 2003). Likewise, yeast eIF4B is associated with robust loading and scanning of PICs and translation of mRNAs and synthetic reporters containing higher than average secondary structure *in vitro* and *in vivo* (Mitchell et al., 2010; Sen et al., 2016). We speculated that the effect of the NTD on translation of mRNAs needed to combat extracellular urea is related to the ability of eIF4B to promote translation of structured mRNAs. To test this hypothesis, we analysed existing parallel analysis of RNA structure (PARS) scores (Kertesz et al., 2010) for the RNAs exhibiting changes in TE as a result of eIF4B NTD deletion, in the presence or absence of urea. PARS scores provide the relative propensity of each nucleotide to a single- or double-strand specific nuclease, with a higher PARS score indicating a higher propensity for secondary structure. Cumulative PARS scores can be compared for specific regions of mRNAs to determine the likelihood that the e.g. first 30 nucleotides, total 5′ UTR, or regions in the ORF have more structure (Figure 4A), which would present an impediment for PIC loading, PIC scanning, or translation elongation, respectively (Sen et al., 2016). A previous analysis reported that deletion of eIF4B in yeast led to reduced translation of mRNAs with long, structured 5′UTRs (Sen et al., 2016). We found that, likewise, deletion of the NTD led to lower translation efficiency (≥1.5-fold decrease) of mRNAs with significantly higher PARS scores for the first 30 nucleotides of the 5′UTR (Figure 4B, First30), (comparison of *∆tif3* and *∆ntd* in Supplementary Figure S10). An even larger difference was observed for the total 5′UTR, suggesting interactions of the eIF4B NTD promote effective mRNA loading and possibly scanning through structured mRNA 5′UTRs (Figure 4B, 5′UTR). The average individual nucleotide PARS score averaged for the full 5′UTRs was likewise significantly higher for these groups of mRNAs that showed reduced TE in the *∆ntd* strain, e.g. presumably higher dependence on the NTD for translation (Figure 4C). In contrast, there was not a significant change in the PARS scores for the 30 nucleotides surrounding the start codon (Figure 4B, Start30), or the first 30 nucleotides of the ORF, suggesting structure around the start site and in the ORF does not strongly require eIF4B activity. In fact, the PARS scores for the Plus45, Plus60 and Plus75 regions were significantly lower for the group of mRNAs that showed higher dependence on the NTD of eIF4B (in the absence of urea). This suggests that the eIF4B NTD is not required for translation of mRNAs with structured ORFs or structured RNAs in general, but instead is important for PIC loading and movement through structured 5′UTRs (Figures 4A,B). The mean length of 5′UTR was also significantly higher for the group of mRNAs that were less efficiently translated (≥1.5-fold) when the NTD of eIF4B was deleted, similar to what was reported for complete eIF4B deletion (Sen et al., 2016). This further suggests the NTD plays a role in effective scanning of PICs through long structured 5′UTRs (Figure 4D). This effect was more pronounced when cells were grown in the presence of 3% urea prior to ribosome profiling, suggesting the effect of the NTD on urea resistance may stem from the ability of eIF4B to promote effective scanning.

We also found that 155 mRNAs showed a relative increase in TE in ∆ntd cells (Figure 3F), indicating ribosomes were able to be loaded on these mRNAs without eIF4B NTD activities. We assessed the degree of secondary structure in the 5′UTRs and coding sequences of these mRNAs (Figures 4E–G, cyan and green) as well as the closed-loop potential. Previous analysis of the tif3∆ mutant demonstrated that mRNAs showing less reliance on eIF4B had shown increased enrichment with components of the closed-loop complex (Sen et al., 2016): eIF4E, eIF4G and PABP (Costello et al., 2015). We likewise compared mRNAs classified as strong-closed loop potential (higher crosslinking immunoprecipitation association with closed-loop components) and no closed-loop potential (enriched in inhibitors of the closed-loop complex(36)) to mRNAs that were translated ≥1.5-fold more efficiently in the *∆ntd* mutant. We found that mRNAs that showed increased TE in *∆ntd* (with or without urea, cyan and green) showed similar trends with respect to PARS scores as those mRNAs defined as having strong closed-loop potential, and the opposite behavior as those mRNAs defined as having no closed-loop potential. Both the Total 5′UTR region and the average per nucleotide PARS scores for the 5′UTRs of these mRNAs showing eIF4B NTD-independence were significantly lower than the average yeast mRNA. In contrast, the mRNAs that showed increased translation efficiency in the ∆ntd mutant and strong-closed-loop associated mRNAs were more structured than the average yeast mRNA in the ORF. This suggests that mRNAs that rely on closed-loop components for mRNA loading do not require the eIF4B NTD or its interaction with the ribosome (Walker et al., 2013), and reinforces the conclusions of the previous manuscript that mRNAs requiring eIF4B activity are less associated with closed-loop components (Sen et al., 2016).

### RNAs Encoding Proteins Trafficked Through the ER and Golgi Have Long and Structured 5′UTRs, Imposing a Heightened Requirement for eIF4B

**FIGURE 5:**
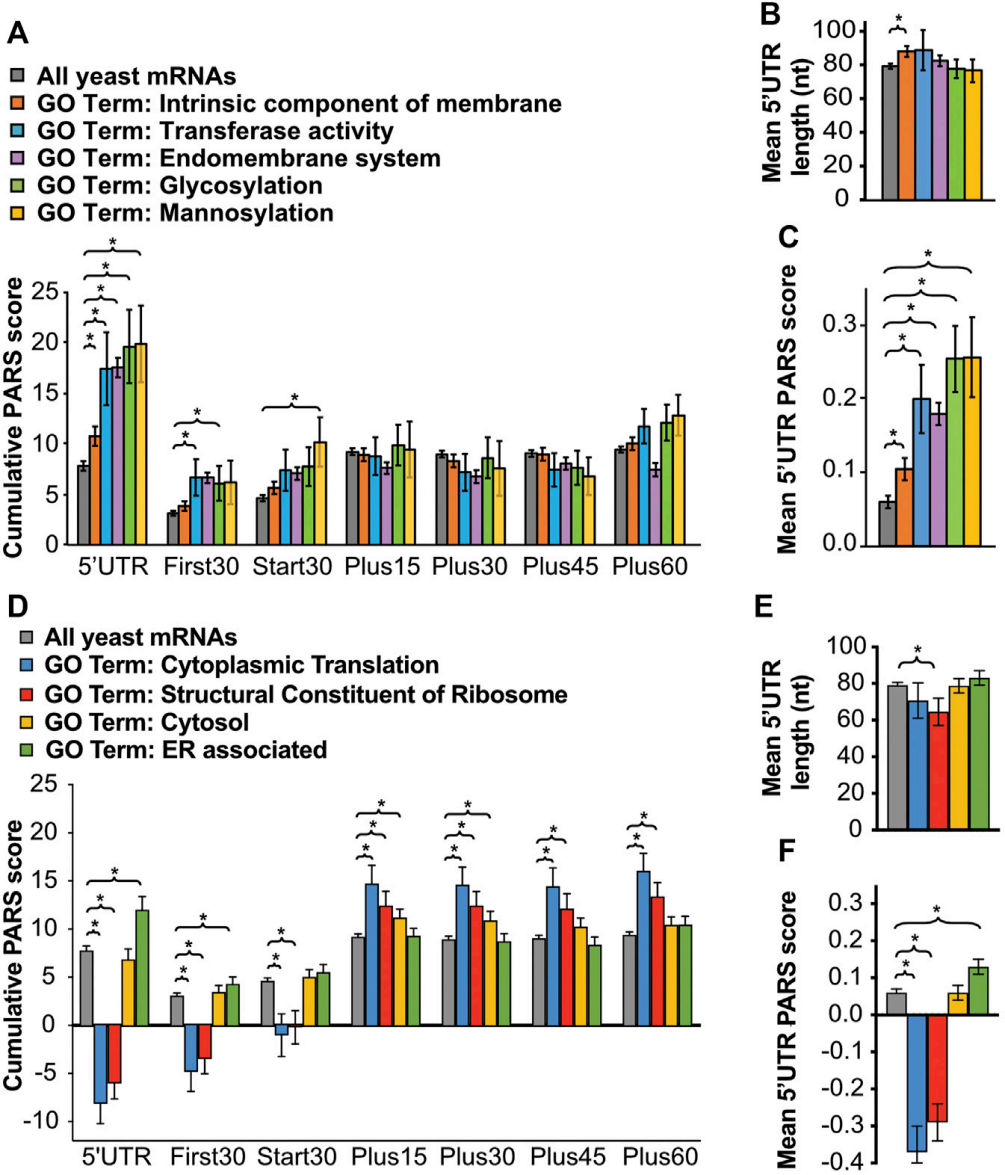
RNAs encoding proteins trafficked through the ER and Golgi have long and structured 5′UTRs, imposing a heightened requirement for eIF4B. Averaged cumulative **(A**,**D)** and single nucleotide **(C**,**F)** PARS scores and 5′UTR lengths **(B**,**E)** for all genes associated with indicated gene ontology categories: intrinsic component of membrane (orange, *n* = 1360), transferase **(A**–**C**, blue, *n* = 86**)**, endomembrane system (purple, *n* = 1098), glycosylation **(A**–**C**, green, *n* = 87**)**, mannosylation **(A**–**C**, yellow, *n* = 46**)**, cytoplasmic translation **(D**–**F**, blue, *n* = 161**)**, structural constituent of ribosome (red, *n* = 190), cytosol **(D**–**F**, yellow *n* = 426**)**, or ER associated **(D**–**F**, green, *n* = 405**)** for each 5′-UTR or CDS interval described as in Figure 4A, with *p* values from Student’s t test indicated (*p < 0.05).

Phenotype microarray analysis suggested the NTD of eIF4B stimulated growth in a number of diverse conditions that challenge cellular integrity (Figure 2A and Supplementary Table S5). The findings that mRNAs translated more effectively by full-length eIF4B had longer and more structured 5′-untranslated regions than those translated when the NTD was deleted led us to question whether proteins for different functions in cells may rely on distinct translational mechanisms. For instance, mRNAs for proteins that promote rapid growth may have less structure and rely less on eIF4B, whereas proteins that allow adaption to stressors, such as the membrane and cell wall proteins, may have more structure and require eIF4B function for translation. If true, the degree of structure would be expected to impose regulatory capacity as cells encounter stresses that require membrane changes, and may explain how the NTD of eIF4B affords resistance to diverse stressors that may require different membrane composition. To investigate this further, we compared the PARS scores for all yeast mRNAs versus the PARS scores for all yeast mRNAs associated with GO terms for mRNAs that required eIF4B for translation in response to urea (Figures 5A–C.) This group includes: intrinsic component of the membrane (the GO term with the lowest *p* value for RNAs showing increased translation in response to urea in WT cells); as well as transferase activity, endomembrane system, glycosylation, and mannosylation (parent GO terms for mRNAs with decreased translation in response to urea in *∆ntd* cells.) Interestingly, we found that the 5′UTRs of mRNAs associated with each of these GO terms had higher average Total PARS scores than the average yeast mRNA (Figure 5A, 5′UTR). However, only those mRNAs encoding intrinsic components of the membrane had significantly longer 5′UTRs (Figure 5B). The mean 5′UTR PARS scores for individual nucleotides was also significantly higher than the average yeast mRNA for all classes (Figure 5C), indicating these classes of mRNAs associated with dependence on the ribosome binding NTD of eIF4B have inherently more structure in the 5′UTRs. This suggests functional importance of structural elements in regulating translation of membrane-associated and trafficked proteins. We then compared the structural composition of mRNAs from two gene ontology categories that were enriched under eIF4B NTD independent translation (Figures 5D–F). We found that as expected, given the observed NTD-independent translation associated with these classes of mRNAs, cytoplasmic translation and structural constituent of the ribosome mRNA categories as whole showed a dearth of structure in their 5′UTRs, with overall negative cumulative PARS scores and mean 5′UTR scores per nucleotide, indicating the 5′UTR regions of these mRNAs are likely to be single stranded. Interestingly, the cytoplasmic translation mRNAs had no significant difference in lengths of their 5′UTRs from the pool of all yeast mRNAs. In contrast to the 5′UTR however, the regions just downstream of the start codon showed higher than average PARS scores for these translation-associated gene ontology classes (Figure 5D, blue and red).

We finally compared the structural content of the two broad gene ontology classes: cytosol and ER. Whereas the cytosol class showed no significant difference in 5′UTR PARs scores from average yeast mRNA, the ER-associated gene ontology class showed significantly higher Total 5′UTR and mean 5′UTR PARS scores than all yeast mRNAs. Moreover, the open reading frames of the ER- associated mRNA pool showed the opposite trend. The cytosol class showed slightly elevated PARS scores for the region immediately downstream of the start site than observed for all yeast mRNAs. Together this suggests higher structure in the 5′UTRs of ER-associated mRNAs than cytosolic mRNAs.

We also took an unbiased approach to exploring the relationship between gene ontology classes, 5′UTR features, and eIF4B NTD-dependence. We ranked all yeast mRNAs based on their cumulative 5′UTR PARS scores (Supplementary Figures S6A–C) or 5′UTR lengths (Supplementary Figures S6D–F) and performed gene ontology analysis to determine enrichment of specific biological processes for the top (B, E) and bottom (C, F) 30% of mRNAs from each group. We compared the degree of overlap between the resulting GO term lists, and found that eIF4B NTD-independence, low 5′UTR structure propensity, and short 5′UTR gene ontology terms showed striking overlap, particularly for the highest enriched GO terms. These mRNAs encode proteins associated with cytoplasmic translation, ribosome biogenesis, and other processes related to ramping up protein synthesis. In contrast, those GO terms enriched for transcripts exhibiting higher NTD-dependence showed some overlap with those enriched in mRNAs with high 5′UTR structure, but considerably less overlap with those enriched for mRNAs with long 5′UTRs. We investigated this relationship further by plotting the log_2_ fold-change in TE as a result of urea and/or NTD-deletion (Supplementary Figure S7). We found that while there was a significant effect correlation of change in TE in urea (for WT or ∆ntd) with 5′UTR lencth, and correlations of 5′UTR PARS with change in TE upon NTD deletion, there may be a threshold level of structure or length at which the NTD becomes necessary to effect change in TE. Overall, these data suggest a complex relationship between the ability of eIF4B to promote translation of mRNAs with structured 5′UTRs and regulation of translation that promotes growth versus regulatory changes.

Finally, we further analyzed the overlap in TE effects for *∆tif3* (Lambert and Draper, 2012) and *∆ntd* strains (Supplementary Figure S8). We found that while there was a correlation between the changes imparted by both mutations, there were also changes in TE that were unrelated between the two mutants. These anticorrelated changes in TE (Supplementary Figure S8D) could be an effect of the seven repeats, or simply an effect of the differences in experimental setup of the previously-published work on the *∆tif3* strain grown at 37°C versus the 30° growth in this work. Interestingly the RNAs showing correlated TEs for the full deletion and ∆ntd have different GO terms than those that are anticorrelated for TE change in the two strains.

## Discussion

In this study, we characterized the contribution of eIF4B RNA- and 40S subunit-binding domains to translational control as well as the ability to promote adaptation of yeast to diverse stressors. We found that the NTD of eIF4B promoted association of eIF4B with PICs and polysomes in yeast while allowing higher TE for RNAs with longer than average and highly structured 5′UTRs, and repression of shorter highly translated mRNAs. These effects were similar to what was observed for deletion of eIF4B. The NTD also afforded higher TE for RNAs encoding proteins trafficked through and modified in the ER and Golgi to reside in cellular membranes. These proteins are expected to remodel the cellular periphery and allow yeast to cope with external stressors.

The RRM of eIF4B was thought to promote mRNA recruitment to ribosomes by providing an RNA anchoring point on a ribosome or eIF3-bound molecule (namely for mammalian eIF4B, (Méthot et al., 1996; Methot et al., 1996; Naranda et al., 1994; Méthot et al., 1994)) or by promoting RNA strand-exchange activities of eIF4B (Niederberger et al., 1998). Our previous work suggested that instead, the RNA-binding activities of the RRM are dispensable for eIF4B function in yeast (Walker et al., 2013). However, because the experiments in our previous work analysed the function of ∆rrm-expressing eIF4B under optimal growth conditions, it remained plausible that the RRM provides additional functions to cells under stress, when additional interactions may be needed to direct ribosomes to specific mRNAs. Our phenotype microarray analysis of the *∆rrm* mutant provides strong evidence that the RRM domain is in fact dispensable for function of this protein in yeast, at least in liquid media. The only plate in which we saw mild phenotypes for the *∆rrm* mutant was in the presence of certain alternative sulfur sources (Figure 2A), but the changes observed were well below the cutoff for significance and were not reproducible. It remains possible that survival in non-vegetative differentiated states could depend on the RRM, and this may explain why the RRM is more important in multicellular organisms (Méthot et al., 1994; Naranda et al., 1994). Alternatively, the contribution of the RRM to cellular processes may not be sufficient to detect a change in growth rate or cellular fitness, but could allow RRM-containing yeast to outcompete mutants defective in RNA-binding. This could have led to retention of the RRM over the course of evolution (Altmann et al., 1993).

**FIGURE 6:**
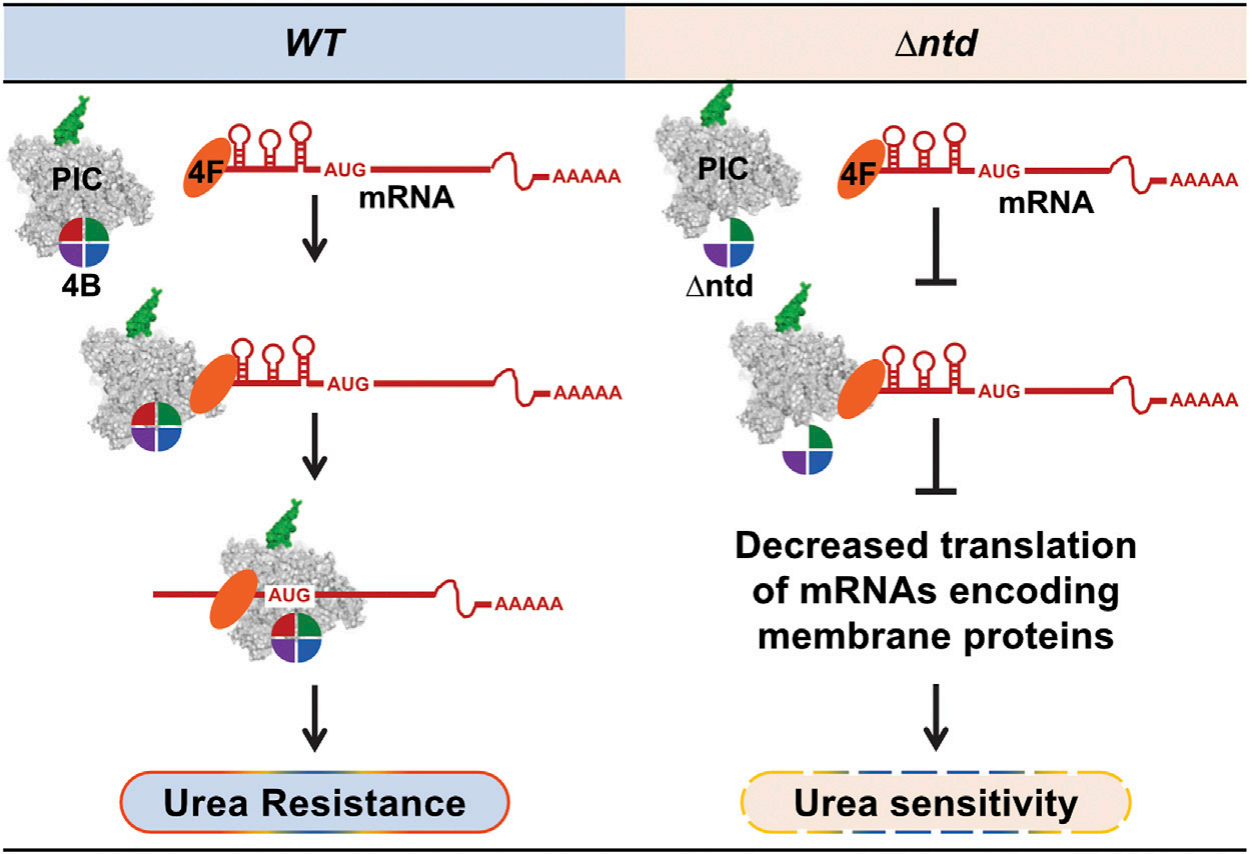
The NTD of eIF4B enhances translation efficiency of mRNAs with structured 5′-UTRs and allows a robust cellular response to urea. WT eIF4B promotes PIC loading and scanning of all mRNAs. Deletion of the NTD of eIF4B reduces translation efficiency of mRNAs with long structured 5′UTRs to a greater extent, indicating eIF4B promotes ribosome loading and scanning while bound to the PIC. Translation of these highly structured mRNAs may be required to reconfigure the membrane proteome and balance translation of cytoplasmic proteins, providing urea resistance.

In contrast to yeast lacking the eIF4B RRM, we found that yeast lacking the NTD were highly sensitive to a number of conditions that WT cells are able to tolerate, and that at least two of these conditions (urea and caffeine) conferred additional changes in translation in the NTD-less mutant (Figure 2 and Supplementary Figure S2, data not shown for caffeine). The mRNAs that showed decreased TE when the NTD was lacking had a number of features similar to those observed for an eIF4B null strain. The 5′UTRs of NTD-dependent mRNAs were longer and more structured than the average yeast mRNA (Figure 4), reinforcing many observations that eIF4B promotes translation of structured mRNAs (Özeş et al., 2011; Dmitriev et al., 2003; Sen et al., 2016; Rogers et al., 2001). The mechanism by which eIF4B is proposed to promote translation of these mRNAs resides in its ability to interact with eIF4A and stimulate helicase activity. However, a report for direct interaction of these factors suggests that the 7-repeats domain of eIF4B binds eIF4A (Andreou et al., 2017). In our study, we observed decreased translation of structured mRNAs when interaction of eIF4B with ribosome complexes was reduced by 80% upon NTD deletion (Figures 1, 4 and Supplementary Figure S1). Related components of the PIC, eIF4A and eIF4G, remained associated with ribosome fractions (Figure 1 and Supplementary Figure S1). This suggests that the mechanism for eIF4B stimulation of structured mRNA translation resides at least to some extent in its ability to bind the ribosome (Figure 6). We have also previously reported defects in functional interaction of eIF4A with eIF4B when either the seven repeats or the NTD is deleted, and observed that overexpression of *∆ntd* has a dominant negative effect on an eIF4A mutant (Walker et al., 2013; Zhou et al., 2014). Together these observations could indicate that deletion of the eIF4B NTD sequesters eIF4A in an inactive state off of the ribosome. However, we did not observe changes in the amount of eIF4A associated with small subunits and translating polysomes when eIF4B occupancy was decreased, arguing against this possibility and suggesting any interactions of the NTD with eIF4A do not drive affinity for ribosome complexes. An alternative possibility is that deletion of the NTD prevents a PIC conformation required for optimal eIF4F activity. In either case, our data suggest the NTD of eIF4B contributes to effective scanning through structured 5′UTRs while bound to the ribosome.

The effect of the eIF4B NTD on recruitment of highly structured mRNAs to the ribosome is in keeping with long-standing models for translational control suggesting that factors which increase the rate of initiation would preferentially benefit mRNAs that are poorly translated (Lodish, 1974). Ribosome profiling data have shown that translation initiation helicases Ded1, Dbp1, and the related human protein DDX3, similarly stimulate translation of specific highly structured mRNAs to a higher degree than unstructured mRNAs that are typically highly translated. Interestingly, despite sharing an ability to promote translation of mRNAs with structured 5′UTRs, analysis of the specific mRNAs that were hyperdependent on the helicases Ded1 (Sen et al., 2015), Dbp1 (Sen et al., 2019), and eIF4A (Sen et al., 2015) showed little overlap with one another or eIF4B, and there was no overlap in the gene ontology enrichment observed for mRNAs hyperdependent on each of these factors. In contrast there was partial overlap between the gene ontology enrichment for mRNAs showing hyperdependence in an eIF4B null strain (Sen et al., 2016) versus the NTD domain deletion that retains some eIF4B activity. This suggests that each of these helicase factors and eIF4B contribute distinct functions to selection of varying classes of mRNAs, perhaps due to varied types and locations of secondary structures.

The strongest NTD-specific growth defect was observed in the presence of urea, which had very little effect on WT or ∆rrm growth rate or translation (P:M ratio) at concentrations that strongly repressed growth and translation of the mutant (Figures 2A–C). Urea affects several processes in *S. cerevisiae*, where it can serve as a nitrogen source, lead to membrane blebbing, and can denature structured nucleic acids. At the concentrations used in this work, it was reported that urea can readily cross the cellular membrane (Cooper and Sumrada, 1975), presumably *via* the Dur3 transporter (Navarathna et al., 2011), and be used as a nitrogen source. Membrane blebbing and denaturation of nucleic acids are unlikely to occur at the ∼0.5 M urea used here (Necas and Svoboda, 1973; Lambert and Draper, 2012). We conclude that translation reprogramming observed in WT cells grown in urea are responsible for growth of those cells at this level of urea. Upon deletion of the eIF4B NTD, the normal translation program is disrupted leading to urea sensitivity.

It is possible that NTD-dependent TE enhancements are needed to produce more of some proteins, or to prevent unregulated derepression of eIF4B-independent mRNAs. Gene ontology enrichment analysis of ≥1.5-fold translation efficiency changes indicated mRNAs encoding proteins associated with the membrane, and to a lesser extent cell wall, showed higher TE in WT cells in response to urea (Figure 3). Likewise, mRNAs encoding proteins associated with endomembrane system and modifications that arise within the ER and Golgi showed decreased TE in response to urea in the mutant cells (Figure 3; Table 1). The resulting membrane proteins are involved in a number of cellular processes. For instance, several paralogous proteins associated with adhesion during a-cell mating (Fig2, Aga1, and Aga2; Supplementary Table S6 and Supplementary Figure S4) showed TE decreases in cells lacking the eIF4B NTD (TE decrease in WT vs ∆ntd in urea of 30-, 5- and 4-fold, respectively, FDR<0.0003). We confirmed an NTD-dependent change in protein level for a *FIG2* 5′UTR driven translation reporter. Recent analyses of uORF usage of yeast cells exposed to temperature shifts indicated that *AGA1* and *AGA2* showed changes in uORF usage in response to temperature shifts (Kulkarni et al., 2019). In the presence of urea, or in response to NTD deletion we did not observe changes in uORF usage of the *AGA2* mRNA. We did not observe ribosome occupancy consistent with translation of the *AGA1* uORF in any of our experiments, and did not observe substantial changes in uORF occupancy overall genome-wide (analysis using uORF seqR not shown, (Spealman et al., 2018)). This suggests the uORF occupancy changes observed in the former study were specific to changes in start codon fidelity in the high temperature response that do not apply in the conditions tested here.

While we found that eIF4B promoted TE of RNAs associated with specific gene ontology classes in response to urea, deletion of the NTD also led to relative increases in TE of RNAs encoding proteins associated with cytoplasmic translation and ribosome biogenesis, which lack structure in their 5′UTRs. In fact, we found substantial overlap in our analysis of gene ontology enrichment for the most unstructured yeast mRNAs (Supplementary Figure S5) and eIF4B NTD-independent gene ontology (Supplementary Tables S7, S8). In some conditions it is likely that derepression of strong closed-loop mRNAs that promote growth could be equally or more detrimental to cells as not producing membrane associated proteins needed for a particular stress response. An analogous scenario has been described for eIF4G-phosphorylation-mediated control of mRNAs in response to glucose starvation in yeast (Chang and Huh, 2018). Reprogramming upon NTD deletion that increases translation of unstructured mRNAs could occur as a result of competition between structured and unstructured mRNA pools for degradation and/or translation machinery. In one scenario, eIF4B may be unable to engage 40S subunits using the NTD to enhance recruitment to structured mRNAs. Ribosomes not being loaded onto eIF4B NTD-dependent mRNAs may be more available to translate mRNAs that do not require eIF4B for ribosome transit through the 5′ UTR. In an alternative scenario, which is not mutually exclusive, increased structured mRNA lacking ribosomes may quench the degradation machinery in RNA granules to prevent proper turnover of housekeeping mRNAs. These additional questions will be of great interest in future work.

## Data Availability

The datasets presented in this study can be found in online repositories. The names of the repository/repositories and accession number(s) can be found below: https://www.ncbi.nlm.nih.gov/geo/, GSE139097.
