# Supplementary material for: Deletion of the N-Terminal Domain of Yeast Eukaryotic Initiation Factor 4B Reprograms Translation and Reduces Growth in Urea
Source: Front Mol Biosci. 2022 Jan 3;8:787781. doi: 10.3389/fmolb.2021.787781 (PMC8762332; doi:10.3389/fmolb.2021.787781)
Supplement: Supplementary file 1 [file DataSheet1.pdf]

Deletion of the N-terminal Domain of Yeast Eukaryotic Initiation Factor 4B Reprograms Translation and Reduces Growth in Urea

Xiaozhuo Liu[1], Houtan Moshiri[1], Qian He[1], Ansuman Sahoo[1], and Sarah E. Walker[1,*]

[1]Department of Biological Sciences, SUNY at Buffalo, Buffalo, NY, 14260, US

* Correspondence: Sarah Walker, walker47@buffalo.edu

SUPPLEMENTARY MATERIALS

Supplementary Table 1: Yeast strains used in this study

Strain Name	Genotype
YSW4	*Mata, his3Δ1, leu2Δ0, ura3Δ0, met15Δ0, tif3Δ0*
YSW5	*Mata, his3Δ1, leu2Δ0, ura3Δ0, met15Δ0, tif3Δ0 [pSW150:HIS3, LEU2, URA3, MET15, TIF3]*
YSW6	*Mata, his3Δ1, leu2Δ0, ura3Δ0, met15Δ0, tif3Δ0 [pSW151:HIS3, LEU2, URA3, MET15, tif3Δntd]*
YSW7	*Mata, his3Δ1, leu2Δ0, ura3Δ0, met15Δ0, tif3Δ0 [pSW152:HIS3, LEU2, URA3, MET15, tif3Δrrm]*
YSW273	*Mata, his3Δ1, leu2Δ0, ura3Δ0, met15Δ0, tif3Δ0 [pSW150:HIS3, LEU2, URA3, MET15, TIF3] [pAS48: KanR FIG2 5'UTR+1st 30-Venus]*
YSW274	*Mata, his3Δ1, leu2Δ0, ura3Δ0, met15Δ0, tif3Δ0 [pSW150:HIS3, LEU2, URA3, MET15, TIF3] [pAS49:KanR, VBA2 P+5'UTR+1st 30-Venus]*
YSW277	*Mata, his3Δ1, leu2Δ0, ura3Δ0, met15Δ0, tif3Δ0 [pSW151:HIS3, LEU2, URA3, MET15, tif3Δntd] [pAS48:KanR, FIG2 P+5'UTR+1st 30-Venus]*
YSW278	*Mata, his3Δ1, leu2Δ0, ura3Δ0, met15Δ0, tif3Δ0 [pSW151:HIS3, LEU2, URA3, MET15, tif3Δntd] [pAS49:KanR, VBA2 P+5'UTR+1st 30-Venus]*

Supplementary Table 2: Plasmids used in this study

Addgene ID	Plasmid Name	Relevant Gene(s)	Markers	Parental Vector	Template or Parts Vectors	Primers
168033	pSW150	*TIF3-His6*	His, Leu, Ura, Met	pHLUM	pFJZ056	SW223/224
168034	pSW151	*tif3Δntd-His6*	His, Leu, Ura, Met	pHLUM	pFJZ141	SW223/224
168035	pSW152	*tif3Δrrm-His6*	His, Leu, Ura, Met	pHLUM	pFJZ140	SW223/224
N/A	pAS39	*FIG2-P-5'UTR-First 30-Venus*	E. coli CamR	pYTK001	BY4741 DNA	AS160/171
N/A	pAS40	*VBA2-P-5'UTR-First 30-Venus*	E. coli CamR	pYTK001	BY4741 DNA	AS164/170
N/A	pAS45	*Intermediate vector for golden gate cloning*	Yeast KanR<br>E. coli AmpR	pYTK003	(pYTK047)<br>(pYTK068)<br>(pYTK077)<br>(pYTK081)<br>(pYTK083)	N/A
168036	pAS48	*FIG2-P-5'UTR-First 30-Venus*	Yeast KanR<br>E. coli AmpR	pAS45	(pAS39)<br>(pYTK045)<br>(pYTK053)	N/A
168037	pAS49	*VBA2-P-5'UTR-First 30-Venus*	Yeast KanR<br>E. coli AmpR	pAS45	(pAS40)<br>(pYTK045)<br>(pYTK053)	N/A

(Parts Vectors for Golden Gate Assembly are indicated in Parentheses)

Supplementary Table 3: Primers used in this study

Primer (cloning)	Sequence
SW223	GGCCGCTCTAGAACTAGTGACCTAATTGACACCGTAC
SW224	CGACGTAGTCGAGGATCGCTTCTTCTTTTGAATATTACC
AS160	TTTCGTCTCGTCGGGGTCTCGAACGCGAGCCTTCCCTTTCAGTA
AS171	TTTCGTCTCGGGTCGGTCTCGAGAACCACTATTCTGGTAGAACACAATTT
AS164	TTTCGTCTCGTCGGGGTCTCGAACGTGGCAACCACATTCTAAG
AS170	TTTCGTCTCGGGTCGGTCTCGAGAACCACCAAGTGCATCAGAAAATG

Primer (RT)	Sequence
ACT1/YFL039C	CGTCTGGATTGGTGGTTCTATC, GGACCACTTTCGTCGTATTCTT
ADH4/YGL256W	TGCTGTCAACGATCCATCTAC, AGAGGCGGTGGAAACATAAG
AGA1/YNR044W	GTAAGTGAAGCCACGAGTACAT CAGACAAGGAGGAGGATGAAAG
AGA2/YGL032C	GAATCGACGCCGTACTCTTT, GGGTGAGAACCGCAATTACT
FIG2/YCR089W	GCAGTGTTGACAGGTTTGTTT, CTTATGGTGGTGGTAGCAGTAG
FLUC	TCATCATGGACAGCAAGACC, CACGAAGTCGTACTCGTTGAA
GCN4/YEL009C	CCAGTTACCACTGACGATGTT, AGTTGTCGAGACTTCCAGATTG
VBA2/YBR293W	CCGAACTGGTTGATTGGTCTAT, GCAGTAGCTTGGTCACTCTTAG
YER186C	GTTTACCAGAGGTGGGTGATAG, CCTCCTTAGTTCTGCACATACA
YOR015W	GCATACTTCAATCGATGTGTCTTC, AAATAGAGGAAAGGCAGAAGGAA

Supplementary Table 4:
Barcode information for RiboSeq and RNASeq library preparation

(attached in a separate file)

Supplementary Table 5:

Stress conditions that significantly affected growth of the eIF4B NTD-deletion strain

Chemical	Panel	Average Height Difference (Δ*ntd* vs WT)
3% Urea	Osmolytes	-129
Trp-Ser	Peptide Nitrogen sources	-113
Trp-Asp	Peptide Nitrogen sources	-112
4% Urea	Osmolytes	-109
Trp-Gly	Peptide Nitrogen sources	-105
Trp-Ala	Peptide Nitrogen sources	-103
Tyr-Ala	Peptide Nitrogen sources	-101
Pro-Gln	Peptide Nitrogen sources	-100
Tyr-Leu	Peptide Nitrogen sources	-97
Lys-Ala	Peptide Nitrogen sources	-97
Thiourea	Phosphorus and Sulfur Sources	-94
2% Urea	Osmolytes	-93
Apramycin sulfate	Chemical Sensitivity	-93
Tamoxifen	Chemical Sensitivity	-93
Phe-Ala	Peptide Nitrogen sources	-92
Chromium (III) chloride	Chemical Sensitivity	-89
L-Citrulline	Nitrogen Sources	-89
Tyr-Gln	Peptide Nitrogen sources	-89
6% Potassium chloride	Osmolytes	-88
Met-Arg	Peptide Nitrogen sources	-87
Ser-Met	Peptide Nitrogen sources	-85
Cystathionine	Phosphorus and Sulfur Sources	-83
Trp-Leu	Peptide Nitrogen sources	-83
L-Cysteine	Phosphorus and Sulfur Sources	-82
Met-Leu	Peptide Nitrogen sources	-82
5% Potassium Chloride	Osmolytes	-82
Cobalt (II) chloride	Chemical Sensitivity	-82
m-Inositol	Nutrient Supplements	-82
Met-Gln	Peptide Nitrogen sources	-81
Poly-L-lysine	Chemical Sensitivity	-81
Thr-Arg	Peptide Nitrogen sources	-80
Caffeine	Chemical Sensitivity	-80
Cysteamine	Phosphorus and Sulfur Sources	-80

Supplementary Table 6:

Comparison of RNAseq, Riboseq and Translation efficiency

(attached in a separate file)

Supplementary Table 7: Gene ontology enrichment for mRNAs with ≥1.5-fold increased translational efficiency in Δ*ntd* in response to urea

GO term	p-Value
Cytoplasm	5.39E-07
Oxidation-reduction process	4.13E-05
Energy reserve metabolic process associated	1.54E-03
Mitochondrion	5.83E-03
Protein refolding	1.24E-02
Mitochondrial envelope	1.94E-02
Response to temperature stimulus associated	2.05E-02
Cellular response to chemical stimulus	2.32E-02
Cellular response to oxidative stress	2.62E-02
Carbohydrate metabolic process associated	3.18E-02
Purine ribonucleoside triphosphate metabolic process	4.45E-02
Oxidoreductase activity associated	4.89E-02

Supplementary Table 8: Gene ontology enrichment for mRNAs with ≥1.5-fold increased translational efficiency in Δ*ntd* without urea

GO term	p-Value
Cytosolic ribosome	1.26E-35
Cytoplasmic translation	2.66E-30
Ribonucleoprotein complex	8.17E-11
Translational elongation	4.44E-10
Organonitrogen compound biosynthetic process	2.99E-08
Cytosol	5.81E-08
Cellular amide biosynthetic process	2.57E-07
Translation	4.16E-07
Peptide biosynthetic process	5.44E-07
Organonitrogen compound metabolic process	2.08E-06
Peptide metabolic process	3.99E-06
Translational frameshifting	2.76E-03
Non-membrane-bounded organelle	3.63E-03
Carboxylic acid metabolic process	7.57E-03

Supplementary Table 9: Gene ontology enrichment for mRNAs with ≥1.5-fold decreased translational efficiency in Δ*ntd* without urea

GO term	p-Value
Intracellular membrane-bounded organelle	5.95E-03
Membrane-bounded organelle	8.32E-03

Supplementary Table 10

Summary of P/M ratio from qRT-PCR for Polysome Gradient Fractions shown in Figure S4 and TE change from Ribosome profiling for selected genes

		qRT-PCR for Polysome Gradient Fractions			Ribosome Profiling	
		P/M (SD Media)	P/M (3% Urea)	Fold change of P/M (3% Urea/SD)	Fold change of TE (3% Urea/SD)	FDR
**AGA1**	WT	1.4	7.25	5.18	4.21	4.10E-04
	Δ*ntd*	0.54	0.46	-1.17	-0.71	4.80E-01
**AGA2**	WT	5.39	7.49	1.39	1.17	NA
	Δ*ntd*	3.43	1.8	-1.91	-3.11	7.63E-03
**FIG2**	WT	0.52	1.18	2.27	15.19	NA
	Δ*ntd*	0.28	0.23	-1.22	-1.60	5.60E-01
**YOR015W**	WT	2.08	3.75	1.80	2.82	NA
	Δ*ntd*	1.18	0.89	-1.33	-1.44	9.10E-01
**VBA2**	WT	0.97	2.03	2.09	1.56	NA
	Δ*ntd*	0.5	0.53	-0.94	-1.45	5.40E-01
**GCN4**	WT	1.12	1.9	1.70	2.66	1.30E-12
	Δ*ntd*	1	1.12	1.12	0.99	9.80E-01
**ACT1**	WT	3.51	5.17	1.47	1.06	9.80E-01
	Δ*ntd*	3.2	3.83	1.20	1.58	3.80E-02
**YER186C**	WT	1.17	1.87	1.60	1.64	NA
	Δ*ntd*	0.66	0.53	-1.25	-11.71	1.30E-01
**ADH4**	WT	1.61	2.6	1.61	1.67	NA
	Δ*ntd*	0.67	0.49	-1.37	-1.04	9.50E-01

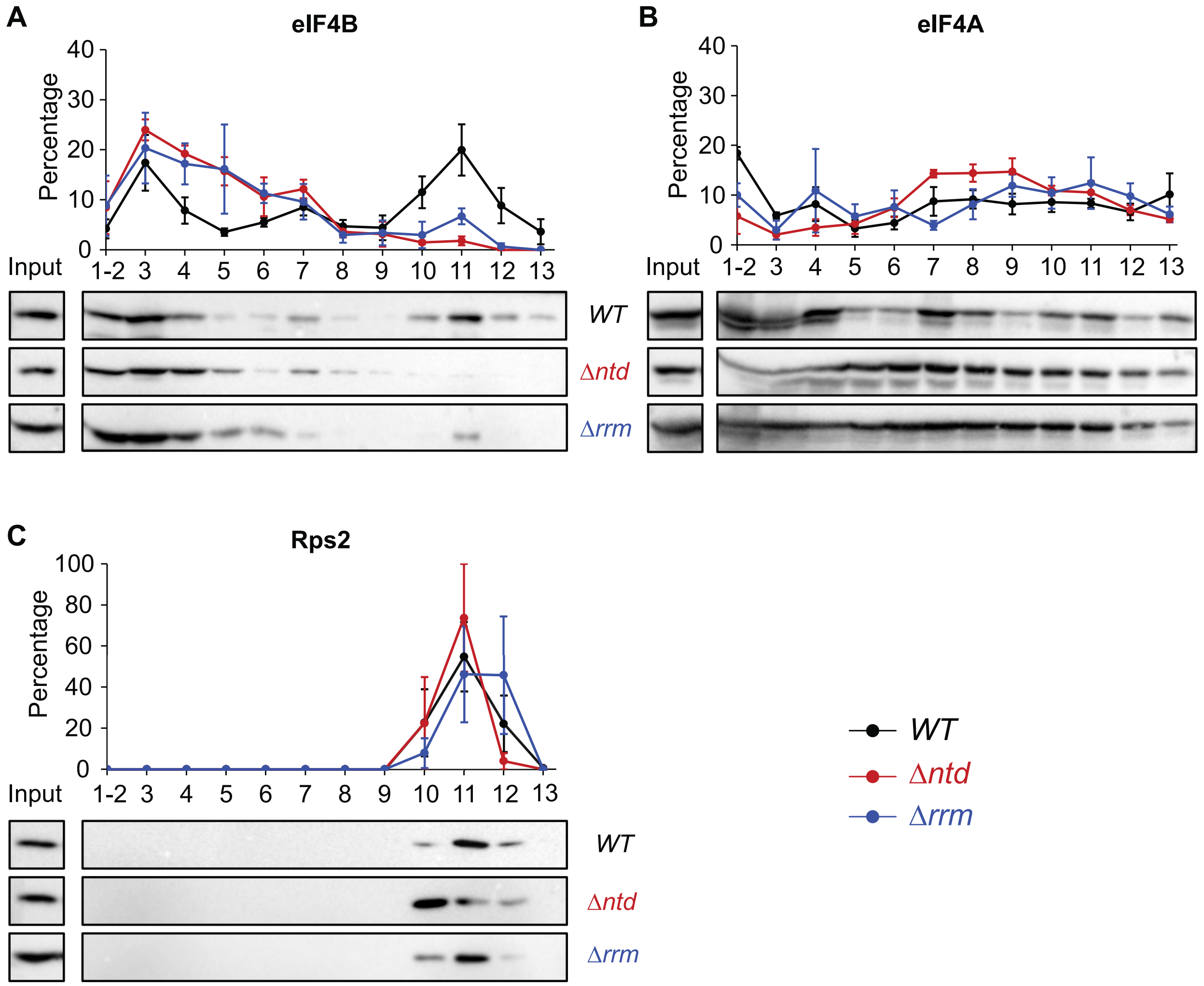

Supplementary Figure 1: eIF4B comigrates with both small subunits and mRNPs in gradients following formaldehyde crosslinking.

Strains harboring WT (black), Δntd (red), and Δrrm (blue) eIF4B were grown in SD media prior to formaldehyde crosslinking. Protein precipitates from lysates were separated on 7.5-30% sucrose gradients and blotted for eIF4B-His6 (A), yeast eIF4A (B), or small subunit protein Rps2 (C).

Supplementary Figure 2

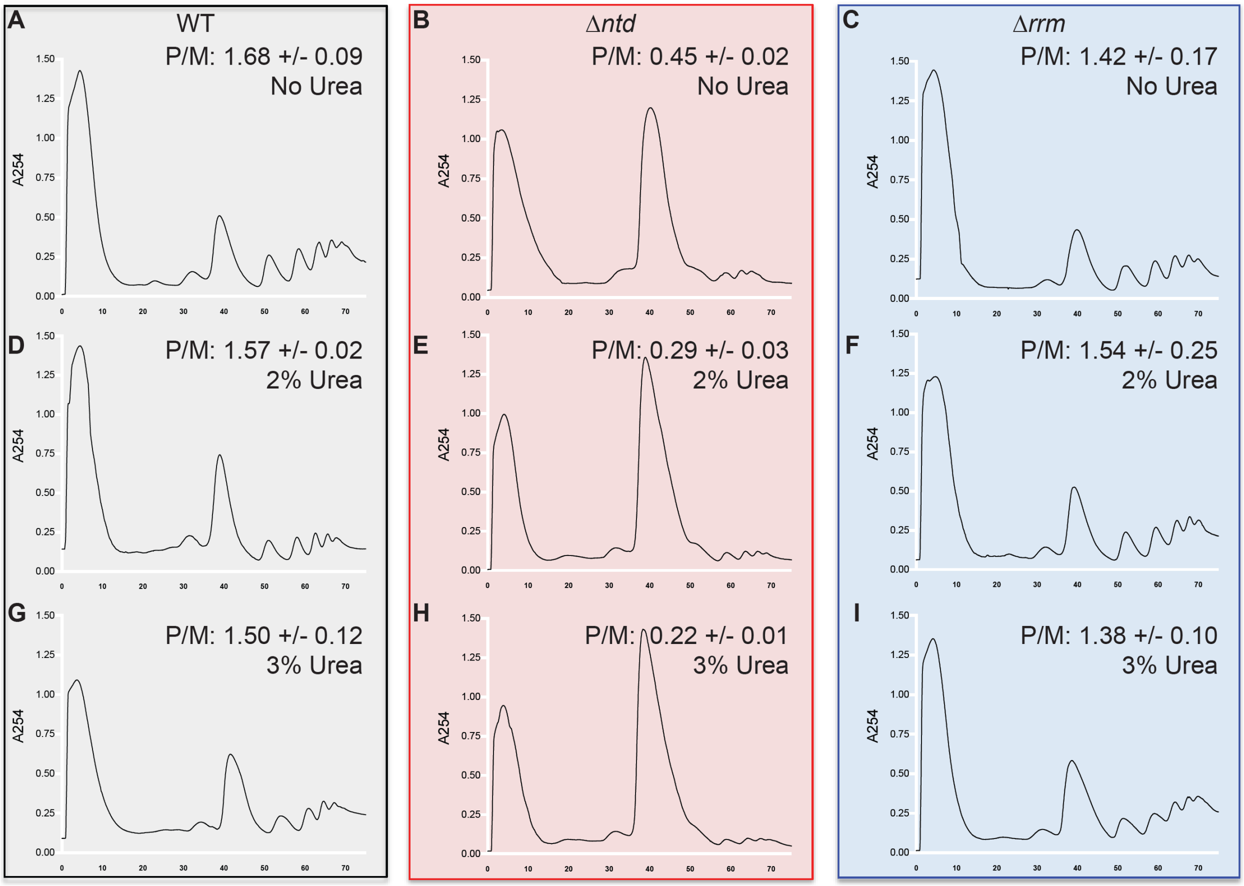

Supplementary Figure 2: Polysome to monosome ratios show both the NTD of eIF4B and Urea conferred changes in the number of ribosomes loaded per mRNA.

Representative sucrose gradient analysis for calculating polysome to monosome changes for no urea (A-C) 2% urea (D-F) and 3% Urea (G-I) in cells harboring WT eIF4B (A, D, G), Δntd (B, E, H), or Δrrm (C, F, I). Deletion of the NTD globally repressed translation, while addition of urea conferred further decreases. The low level of translating ribosomes in the Δntd mutant makes the increase in monosomes upon urea addition more apparent by eye than the reduction in polysomes.

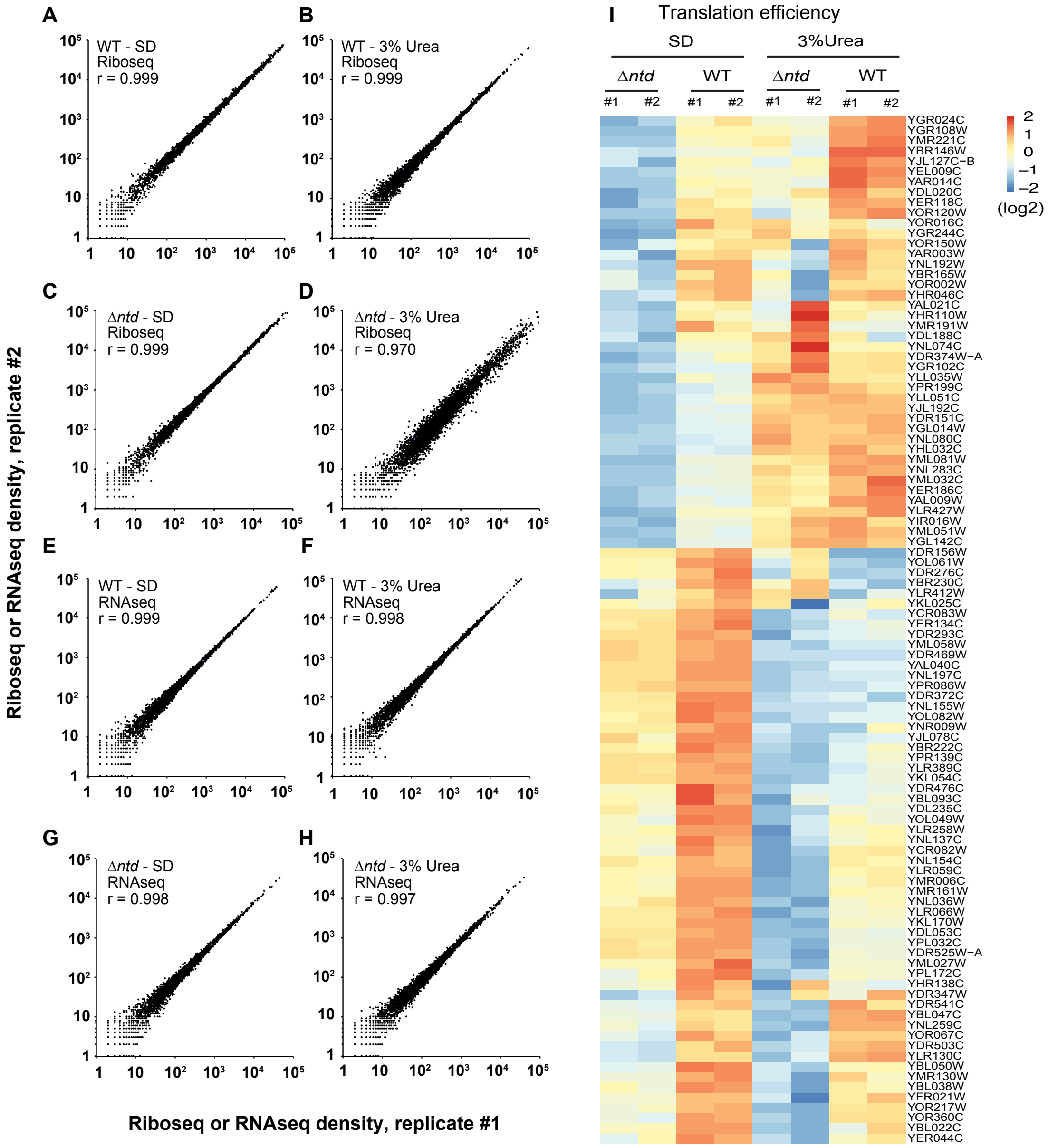

Supplementary Figure 3: Riboseq and RNAseq reads are reproducible for independent replicates and show clustered changes in TE in response to urea and NTD mutation. Riboseq (A-D) and RNAseq (E-H) densities on individual mRNAs were compared for two biological replicates of WT and NTD deletion mutants with and without urea to determine reproducibility of samples. Pearson correlation coefficients (r) were calculated from each comparison plot. (I) Heatmap of differential translation efficiencies (TE) for mRNAs with largest TE change in NTD deletion mutant as compared to WT.

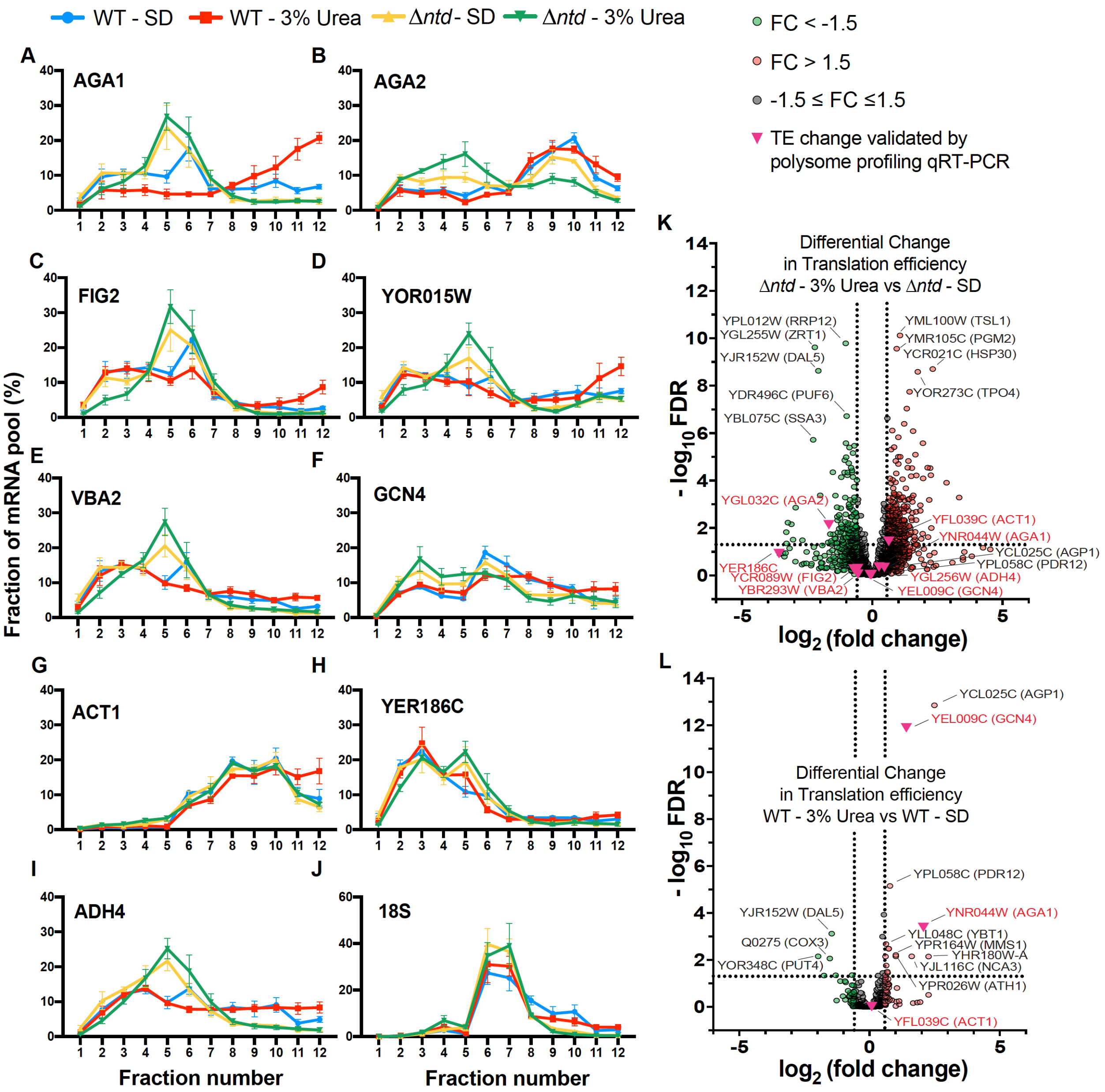

Supplementary Figure 4

Supplementary Figure 4: Deletion of the NTD of eIF4B and exposure to urea promote TE changes. A-J qRT-PCR of gradient fractions for: WT without urea (blue), WT with 3% urea (red), Δ*ntd* without urea (yellow), and Δ*ntd* with 3% urea (green). Values were normalized to RNA spike-in control in each fraction and plotted as percentage of RNA in all fractions. Results from three biological replicates ± SEM are shown. The mRNAs showing increased association with polysome-containing fractions (Fractions 8-12) in WT in response to 3% urea (red) are: *AGA1*, *FIG2*, and *YOR015W* (note: *FIG2* and *AGA1* are paralogs); while genes showing decreased association with polysome-containing fractions as a result of NTD-deletion (green and yellow) are *AGA1*, *FIG2*, *YOR015W*, *VBA2*, and *ADH4*. *AGA2* shows decreased polysome association upon NTD deletion and further modest decreases in urea. 18S rRNA shows decreased levels in polysomes as a function of urea and the NTD, and verifies 40S and ribosome location. (K-L) Volcano plots showing urea-dependent differential changes of translation efficiency in NTD deletion mutant (K) and in WT (L).

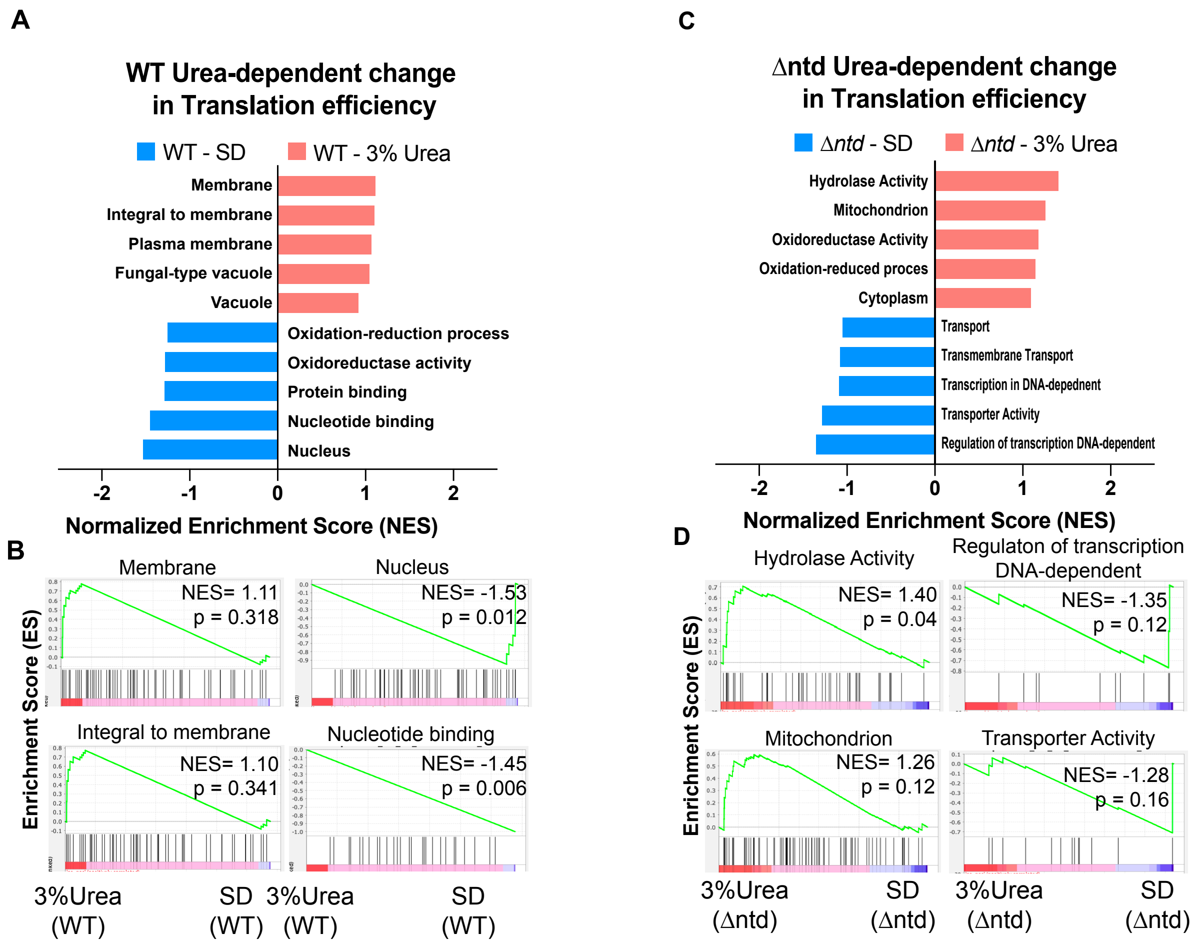

Supplementary Figure 5: Gene set enrichment analysis (GSEA) on ranked lists of mRNAs based on their TE change in response to 3% urea in WT (A, B) and NTD-deletion mutant (C, D). (A, B) GSEA showing enrichment of membrane, integral to membrane, plasma membrane gene signature in WT in response to 3% Urea, and enrichment of nucleus, nucleotide binding in WT in SD condition. (C, D) GSEA showing enrichment of hydrolase activity, mitochondrion gene signature in NTD-deletion mutant in response to 3% Urea, and enrichment of regulation of transcription DNA-dependent, transporter activity in NTD-deletion mutant in SD condition.

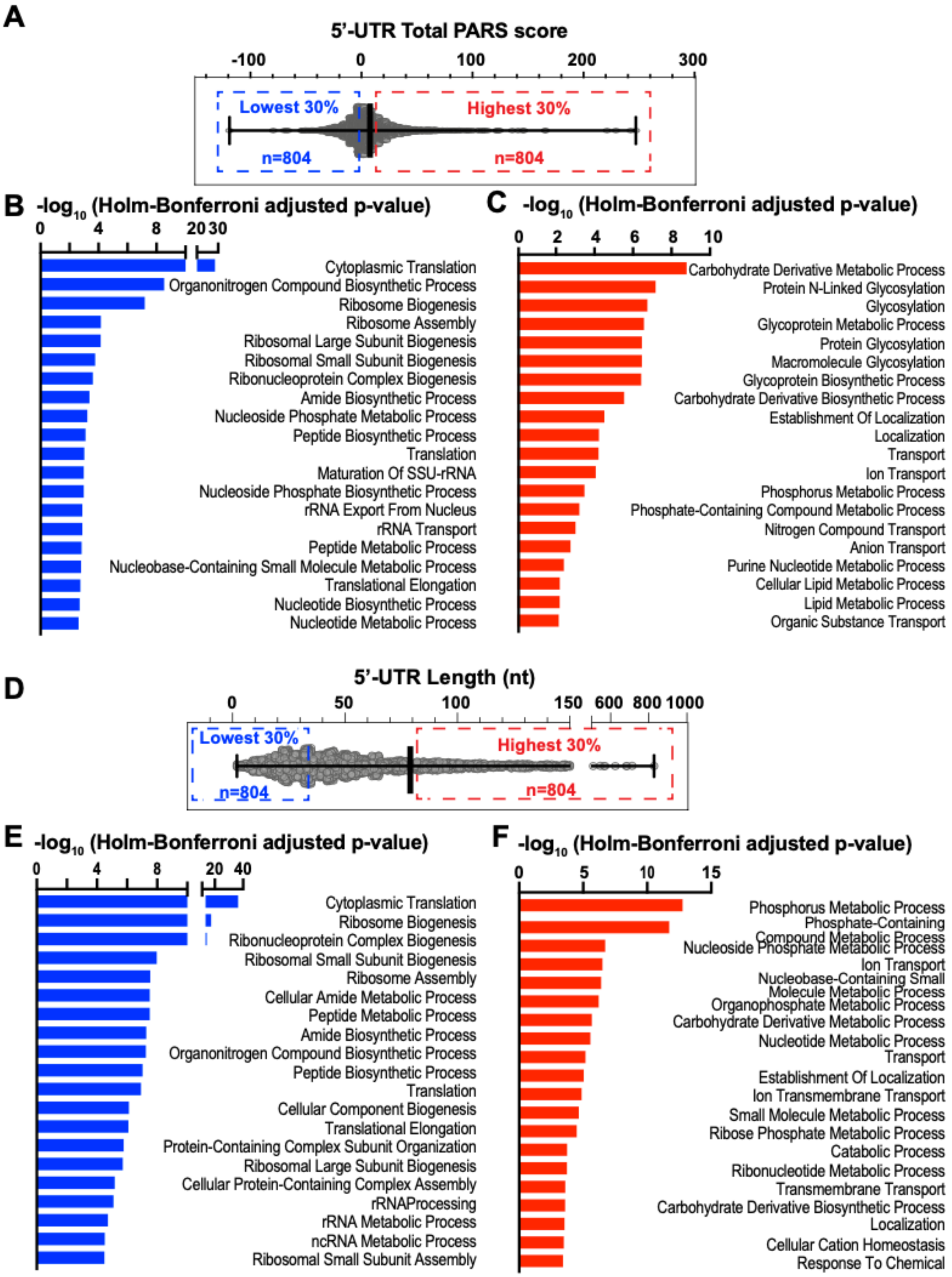

Supplementary Figure 6: Gene ontology analysis for yeast mRNAs grouped based on 5'UTR length (D-F) and propensity for involvement in secondary structure (A-C.) Total 5'UTR PARS scores (A) and Lengths of 5'UTRs (D) for all yeast mRNAs were ranked, and the 30% highest (C, F) and lowest (B, E) for each ranking were analyzed for gene ontology enrichment at Yeastmine.

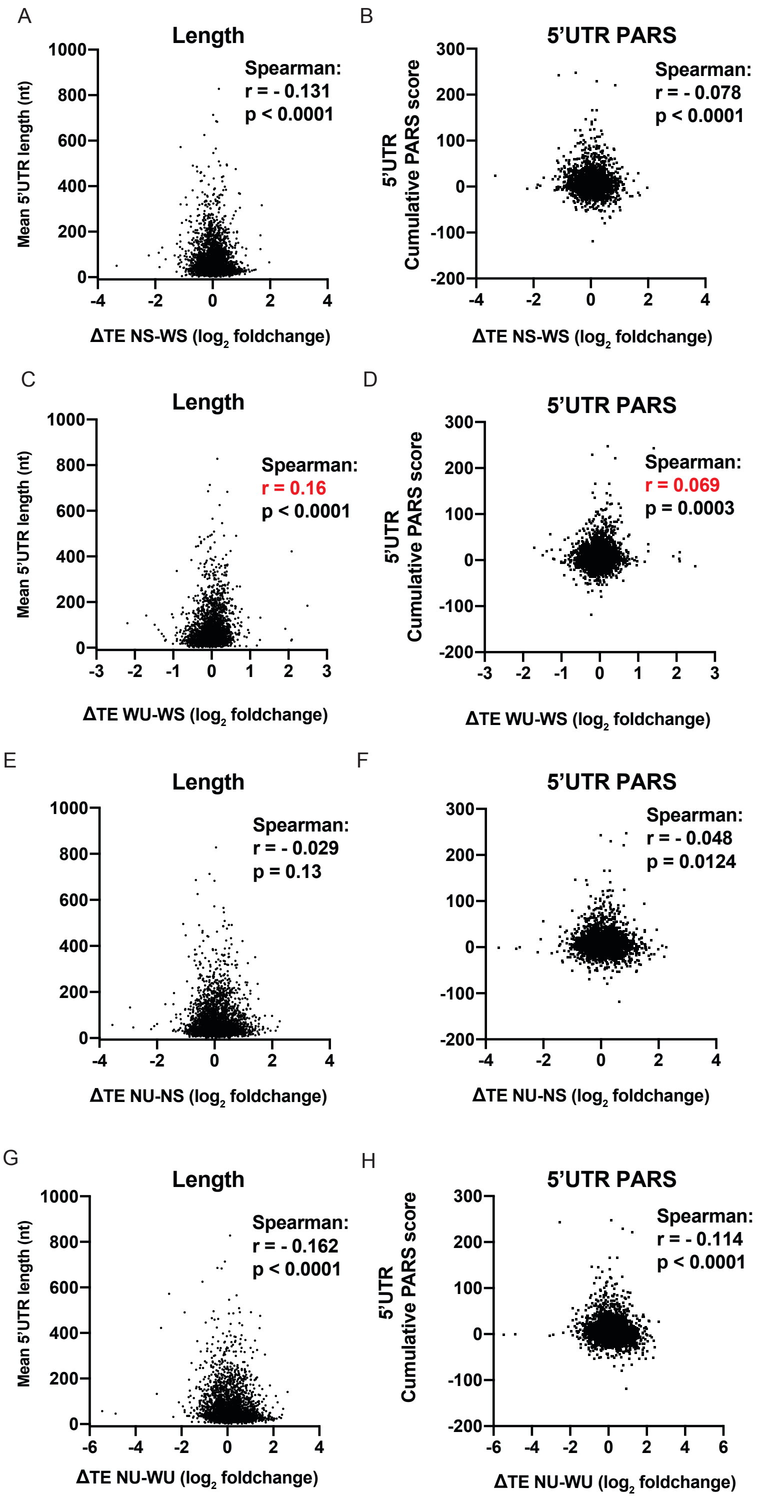

Supplementary Figure 7: Correlation of 5'-UTR length and TE change in Δ*ntd* vs. *WT* in SD media (NS-WS) (A), or in *WT* 3% Urea vs. *WT* SD condition (WU-WS) (C), or in in Δ*ntd* 3% urea vs. Δ*ntd* SD condition (NU-NS) (E), or in Δntd vs. WT in 3% urea. (G). Correlation of 5'-UTR PARS score and TE change in Δntd vs. WT in SD media (NU-WU) (B), or in WT 3% Urea vs. WT SD condition (D), or in in Δntd 3% Urea vs. Δntd SD condition (F), or in Δntd vs. WT in 3% urea. (H).

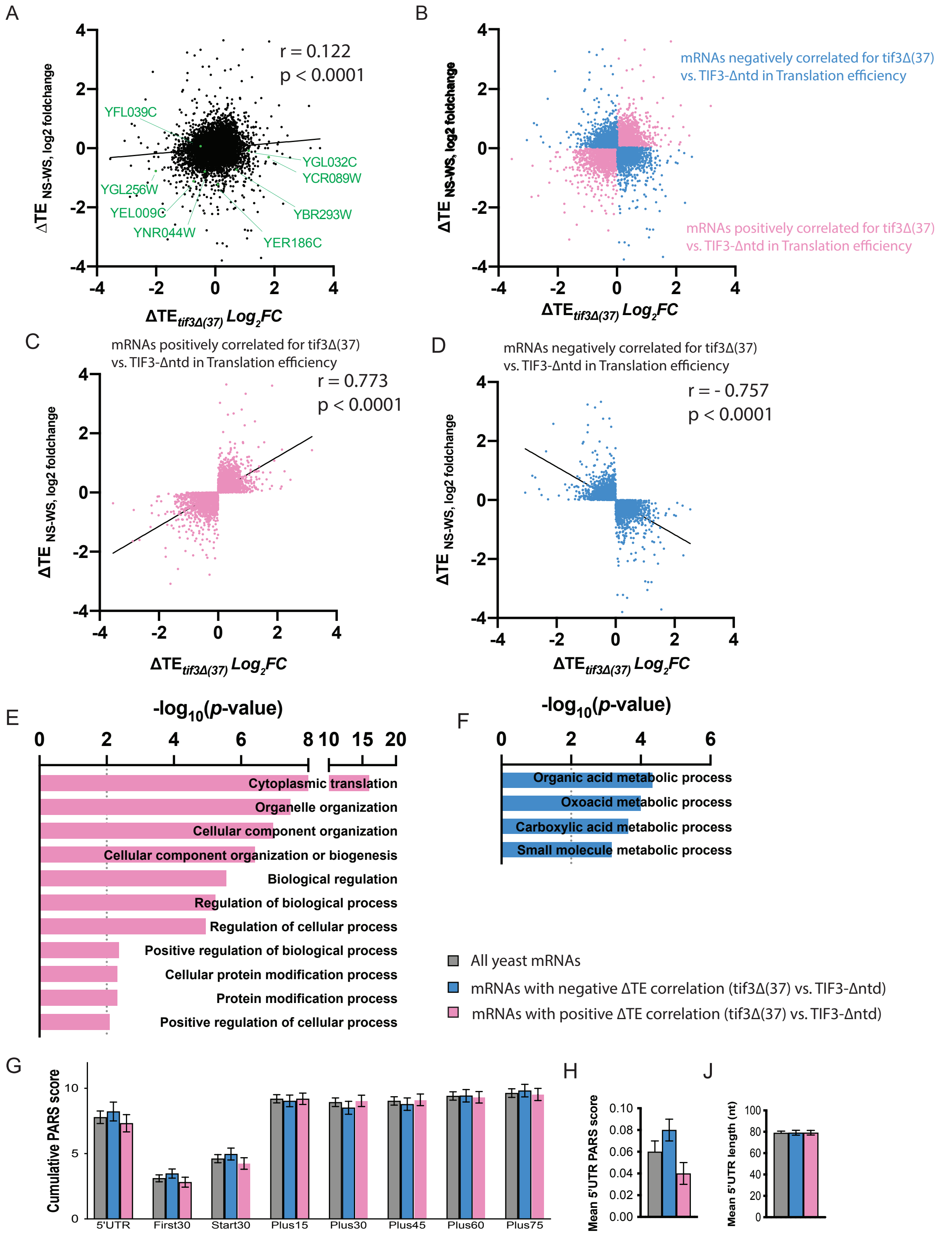

Supplementary Figure 8: Correlation of TE change in *tif3*Δ(37) versus TE change in Δ*ntd*. (A) Scatter plot showing the correlation of TE changes in *tif3Δ(37)* and Δ*ntd*. Pearson correlation coefficient is shown. (B) RNAs were further divide into two groups: (C) RNAs showing correlated changes in TE in *tif3*Δ(37) vs. Δ*ntd*; and (D) RNAs anticorrelated for change in TE in *tif3*Δ(37) vs. Δ*ntd*. PARS (G-J) and Gene ontology analysis for correlated(E) and anticorrelated (F) RNAs.
